# Topotactic fluorination of intermetallics as an efficient route towards quantum materials

**DOI:** 10.1038/s41467-022-29043-8

**Published:** 2022-03-18

**Authors:** Jean-Baptiste Vaney, Baptiste Vignolle, Alain Demourgues, Etienne Gaudin, Etienne Durand, Christine Labrugère, Fabio Bernardini, Andrés Cano, Sophie Tencé

**Affiliations:** 1 CNRS, Université Bordeaux, Bordeaux INP, ICMCB, UMR 5026, Pessac, France; 2 CNRS, Univ. Bordeaux, PLACAMAT UMS 3626, Pessac, F-33600 France; 3 Dipartimento di Fisica, Università di Cagliari, IT-09042 Monserrato, Italy; 4 CNRS, Université Grenoble Alpes, Institut Néel, 38042 Grenoble, France

**Keywords:** Reactive precursors, Solid-state chemistry, Superconducting properties and materials

## Abstract

Intermetallics represent an important family of compounds, in which insertion of light elements (H, B, C, N) has been widely explored for decades to synthesize novel phases and promote functional materials such as permanent magnets or magnetocalorics. Fluorine insertion, however, has remained elusive so far since the strong reactivity of this atypical element, the most electronegative one, tends to produce the chemical decomposition of these systems. Here, we introduce a topochemical method to intercalate fluorine atoms into intermetallics, using perfluorocarbon reactant with covalent C-F bonds. We demonstrate the potential of this approach with the synthesis of non-stoichiometric mixed anion (Si-F) LaFeSiF_x_ single-crystals, which are further shown to host FeSi-based superconductivity. Fluorine topochemistry on intermetallics is thus proven to be an effective route to provide functional materials where the coexistence of ionic and metallo-covalent blocks, and their interactions through inductive effects, is at the root of their functional properties.

## Abstract (web summary)

Insertion of light elements in intermetallics has been explored to synthesize functional materials. Here the authors report topotactic intercalation of fluorine atoms into intermetallics using a perfluorocarbon reactant with covalent C-F bonds to obtain quantum materials.

## Introduction

A large number of topotactic reactions, preserving the architecture of the crystal lattice, have been demonstrated since the 1970s. In a topochemical phenomenon, intercalation or deintercalation of an ion is compensated by electron transfers to maintain charge neutrality. Within complex transition metal-oxide networks, this results in the reduction/oxidation of the transition elements M (mainly M = Mn, Fe, Co, or Ni). The intercalation of polarizing cations such as Li^+^ in lamellar nickel-based oxides —from Li_1−*x*_MO_2_ to LiMO_2_^1^— or the removal of O^2−^ ions in perovskite-type networks provides two important examples widely mentioned in the literature. In the latter case, the phenomenon leads to the stabilization of infinite layers of Fe^2+^ (3d^6^) (from SrFeO_3−*x*_ to SrFeO_2_^2^) and also Ni^+^ (3d^9^) ions in an unusual electronic configuration (from Nd_1−*x*_Sr_*x*_NiO_3_ to Nd_1−*x*_Sr_*x*_NiO_2_^3^) with a well-marked two-dimensional character. Combined to thin film growth techniques, these methods applied to nickelates have recently started a new chapter in the field of superconductivity^3^.

In all these new compounds based on a 2D architecture, the alternation of layers whose chemical bonding harbors very different natures, are key features. In fact, the coexistence and interplay between ionic and covalent bonds together with Van der Waals forces emerge as the common thread among these systems where topochemical reactions occur. One illustration of it is the so-called inductive effect^4^, according to which the more covalent the chemical bonding within the covalent layers, the more ionic character the bonds between these layers. Thus, the topochemical approach allows not only to obtain novel compounds with new types of functional blocks but also to fine-tune some of their physical properties by playing with the nature of the chemical bonds. In fact, most of these topotactic systems exhibit mixed ionic-electronic conductivity. However, to obtain novel materials by directly stacking layers of ionic and metallic (or covalent) character remains a fundamental challenge.

**Fig. 1:**
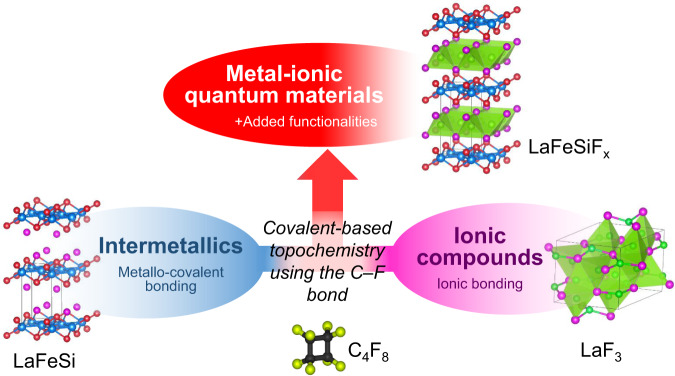
Chemical synthesis strategy. Strategy to provide metal-ionic compounds based on topochemical reaction of perfluorocarbon reactant on an intermetallic compound as a host.

Intermetallics is an important family of materials that naturally involves metallic and covalent bonding. Layered intermetallics are especially good candidates to design new compounds via this topochemical strategy by intercalating the appropriate species (element or molecule). However, when considering ionic and covalent bonds in halides, oxides, or chalcogenides where topochemical reactions assisted by redox phenomena take place, intercalation process is more difficult to handle in intermetallics which harbor extensive electronic delocalization. Among possible intercalated species, the strongly electronegative fluoride anion stands as an interesting candidate to promote novel properties in the overall structure, owing to its ability to alter chemical bonding through inductive effects and thereby creating ionic blocks. Fluorine insertion, however, has remained a challenge in intermetallic compounds. Unsuccessful attempts^5^ are ascribed to chemical decomposition caused by the over-reactivity of the hard base F^−^ anion on hard acid cations according to Pearson’s theory. Yet, overcoming this challenge would open the door to the coexistence of metallic-covalent and highly ionic blocks within the same structure, thereby promoting a new class of materials with potentially new electronic properties (Fig. 1).

**Fig. 2:**
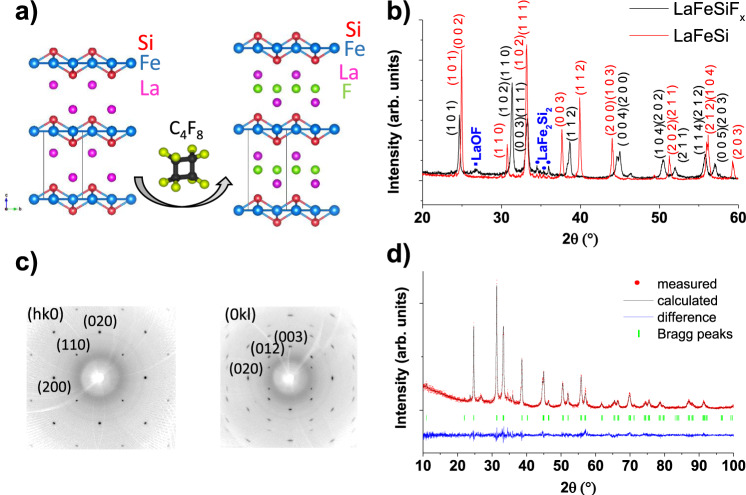
X-Ray diffraction. **a** Illustration of the LaFeSi fluorination upon C_4_F_8_ treatment, after which the F atoms are inserted into the 2*b* Wyckoff positions of the *P*4/*nmm* structure. **b** Indexed powder X-ray diffraction patterns of LaFeSi (*a* = 4.11 Å, *c* = 7.16 Å) and LaFeSiF_*x*_ (*a* = 4.03 Å, *c* = 8.09 Å). The successful fluorination of the LaFeSi intermetallic is clearly evidenced through the shift of the Bragg peaks position. **c** Indexed precession images of the single-crystal X-ray diffraction pattern of a 50 × 50 × 10 µm^3^ LaFeSiF_0.2_ crystal (*a* = 4.05 Å, *c* = 8.11 Å) corresponding to (hk0) and (0kl) family planes of the reciprocal space. **d** Rietveld refinement of the LaFeSiF_*x*_ powder sample. All structural parameters were refined except the F content x whose accuracy is not sufficient from powder data.

Here, we introduce a topotactic fluorination method that enables the synthesis of fluorinated intermetallics, including non-stoichiometric single-crystals. The method is based on the use of c-C_4_F_8_ perfluorocarbon reactant whose decomposition into CF_2_ radicals with high dissociation energy allows reducing sufficiently the reactivity of fluorine. F^−^ can then slowly diffuse through the material without decomposing it. Thus, we use this method to achieve the proof-of-principle intercalation of fluorine into the LaFeSi intermetallic compound, which yields the series of non-stoichiometric LaFeSiF_*x*_ (see Fig. 2a). In addition, we show the emergence of superconductivity across this series which thus extends the family of Fe-based superconductors to FeSi-based materials beyond the conventional ferropnictides and chalcogenides.

## Results and discussion

### Topochemical synthesis of non-stoichiometric fluorosilicides and characterization

We took LaFeSi as a model system to illustrate the topotactic fluorination of intermetallic compounds. This particular system is a simple Pauli paramagnet that belongs to the rich family of 111-type layered intermetallics (*RTX* with *R* = rare earth, *T* = transition metal, and *X* = *p*-block element)^6^. As shown in Fig. 2a, FeSi layers are separated by a double layer of La atoms left with empty tetrahedral sites. Direct F intercalation into the La spacer of LaFeSi was successfully achieved by means of a mild topotactic fluorination technique that enables the controlled release of over-reactive F^−^ ions. Specifically, we treated the LaFeSi powder with gaseous octafluorocyclobutane c-C_4_F_8_ at 500 °C. This produces a mixture of two fluorinated phases. These phases are free from LaF_3_, and have only a tiny amount of LaOF (Supplementary Figs. 1 and 2). Subsequent annealing at 500 °C homogenizes the F content within the sample. Thus, we finally obtained nominally pure and well-crystallized fluorinated phases isostructural to the reference 1111 materials LaFeAsO and LaFeSiH^7^. The corresponding X-ray diffraction (XRD) pattern is illustrated in Fig. 2b–d. When subjecting LaFeSi single crystals to the same process, well-ordered and homogeneous single crystals of LaFeSiF_*x*_ are obtained, as demonstrated by precession images of a single-crystal LaFeSiF_0.2_ XRD pattern (Fig. 2c). This achievement indicates a direct fluorination of an intermetallic, both in the powder and single crystal form. The successful homogeneous diffusion of fluoride anions within the structure at medium temperature and over millimetric distances for single crystals strongly indicates ionic conduction of the fluoride anions. We recall that most of complex fluorides, adopting non-stoichiometric fluorite or tysonite-type structures for instance, exhibit high fluoride (F^−^) ion conductivity at room temperature with low activation energy (5 × 10^−4^ S cm^−1^, *E*_a_ = 0.30 eV in Ce_1−*x*_Sr_*x*_F_3−*x*_^7^) which reflects a high mobility of F^−^ ion. The ionic conductivity is strongly affected by the concentration of fluorine vacancies and consequently by the chemical composition^8^. We note that the LaF blocks in LaFeSiF_*x*_ network derive from the fluorite-type structure.

**Fig. 3:**
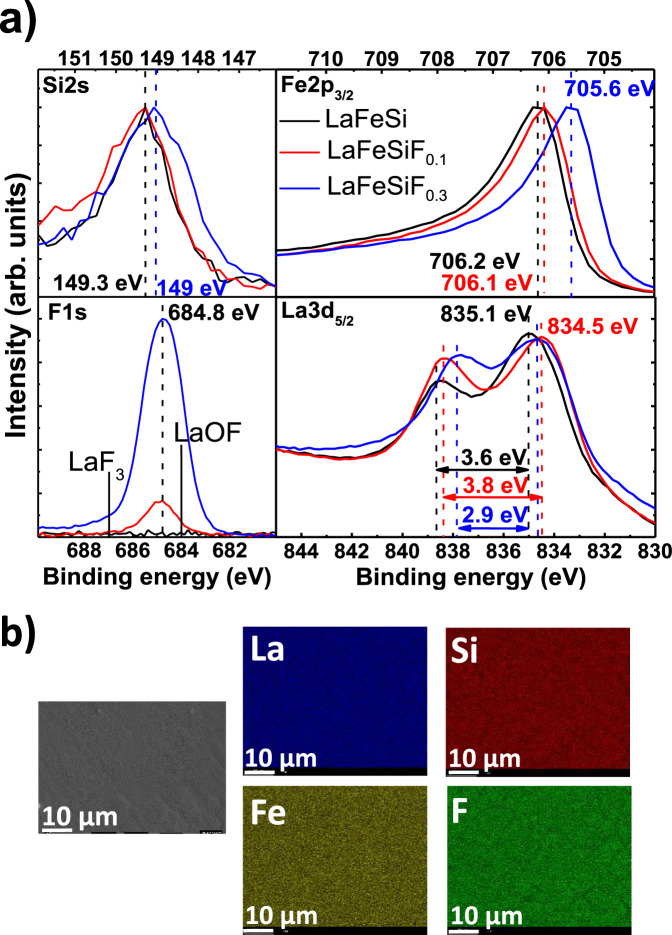
X-Ray photoelectron spectroscopy. **a** XPS signals on the surface for LaFeSiF_*x*_ (*x* = 0, 0.1, 0.3) single crystals, for the Si2*s* F1*s*, Fe2*p*_3/2_, and La3*d*_5/2_ core shells. The photoemission signals were acquired after several etching steps up to ~100 nm depth. **b** Secondary electron picture of the surface of a single-crystal LaFeSiF_0.3_ and corresponding La, Fe, Si, and F distribution maps for the same spot as determined by EDS analysis. The probed depth of the crystal is typically 1 µm.

We further demonstrated the incorporation of fluorine in LaFeSi by means of X-ray photoelectron spectroscopy (XPS, Fig. 3a) and Energy Dispersive Spectroscopy (EDS) techniques (Fig. 3b). We measured three single crystals with XPS: LaFeSiF_0.3_, LaFeSiF_0.1_, and LaFeSi (the exact F amount is determined from the linear relationship between the F content *x* and the c-axis parameter for *x* > 0.09, as shown later by the analysis of single-crystal XRD data). As shown by EDS analysis on LaFeSiF_0.3_, the four elements are clearly homogeneously distributed within the crystals. Besides, upon etching of the surface, we obtained an XPS signal more representative of the bulk at La 3*d*, Fe 2*p*, Si 2*s*, and F1*s* energy ranges. The F1*s* peak appears clearly ~685 eV at the same energy position whatever the F content. However, the intensity of the F1*s* peak increases from LaFeSiF_0.1_ to LaFeSiF_0.3_ compounds as well as the FWHM (Full Width at Half Maximum) value, associated to the heterogeneous distribution of the fluorine atoms in LaF sheet. The binding energy of the F1*s* differs from the energies typically observed in LaF_3_ and LaOF (respectively 687 eV^9^ and 684 eV^10^), once again confirming the bulk character of the F insertion. Furthermore, the energies and shapes of the La3*d*_5/2_ main peak ~835 eV and the satellite peak ~838 eV strongly change upon fluorine insertion. The relative intensity of the satellite peaks depends on the orbital overlap between La atoms and its ligand atoms (F and Si). We ascribe the large increase in intensity of the satellite and its energy shift relative to the main peak upon F intercalation to a more pronounced overlap between La and Si atoms, given that the binding energy of the F1*s* remains constant^11,12^. Finally, on the Si2*s* and Fe2*p*_3/2_ sides, a clear decrease of binding energy is evidenced after F incorporation, thereby illustrating an enhancement of the covalency of the global Fe-Si chemical bonding. Such analysis reveals valuable information showing that after topochemical fluorination orbital overlapping within FeSi block and that involving La-Si chemical bonding both increases noticeably. While the covalent character increase in the FeSi sheet was clearly expected, due to the inductive effect caused by the ionicity of the LaF block, the same effect observed for the La-Si bond was somewhat unexpected but can still be ascribed to the influence of the ionic La-F bond. The creation of a low content of La-F ionic bonds (10% occupancy in the Td site of La) enhances the sharing electrons around the most polarizable anion Si involved in more covalent bond with polarizable La and polarizing Fe.

### Reactional mechanism

In addition, we also treated both LaFeSi and LaFeSiH with several conventional fluorinating agents (F_2_, NH_4_F, KF, CF_4_, PVDF_…_), but none of these methods (or high-temperature direct synthesis) yielded LaFeSiFx, nor avoided the formation of the very stable ionic binary fluoride LaF_3_ (Supplementary Figs. 3–8). This emphasizes the efficiency of our fluorination route with c-C_4_F_8_. We also obtained similar results on powder with the use of polytetrafluoroethylene (PTFE) precursor (but with significant LaF_3_ amount) (Supplementary Fig. 9). This suggests a tentative reactional mechanism at play between LaFeSi and c-C_4_F_8_, as PTFE thermally decomposes into the same radicals as this gas^13^. c-C_4_F_8_ is a perfluorocarbon gas mostly relevant in its use in surface treatment in the semiconducting industry, or for its insulating properties^14^. In our case, the effectiveness could lie in the decomposition of the cyclic perfluorocarbon, first into tetrafluoroethylene molecules C_2_F_4_ at 500 °C and later into difluorocarbene (:CF_2_) radicals^15^, as observed locally in c-C_4_F_8_ plasma treatments on Si^16^. These electron-deficient :CF_2_ radicals, presenting an unfilled *p* orbital, should naturally be attracted towards the electron-rich LaFeSi. An adsorption reaction of CF_2_ radicals combined to their high dissociation energy (210 kcal/mol^17^ compared to 40 kcal/mol for F_2_) kinetically allows for a slow release of F^-^ ions and their subsequent diffusion into the structure. In this view of the mechanism, fluorocarbon reactants or elemental carbon should also appear as co-products. Although we were not able to identify fluorocarbon co-products, X-ray photon spectroscopy provided a spectrum for the 1*s* levels of carbon (Supplementary Fig. 13) suggesting the presence of graphene-like layers of carbon on the LaFeSiF_*x*_ surface after treatment.

### Structural analysis

**Fig. 4:**
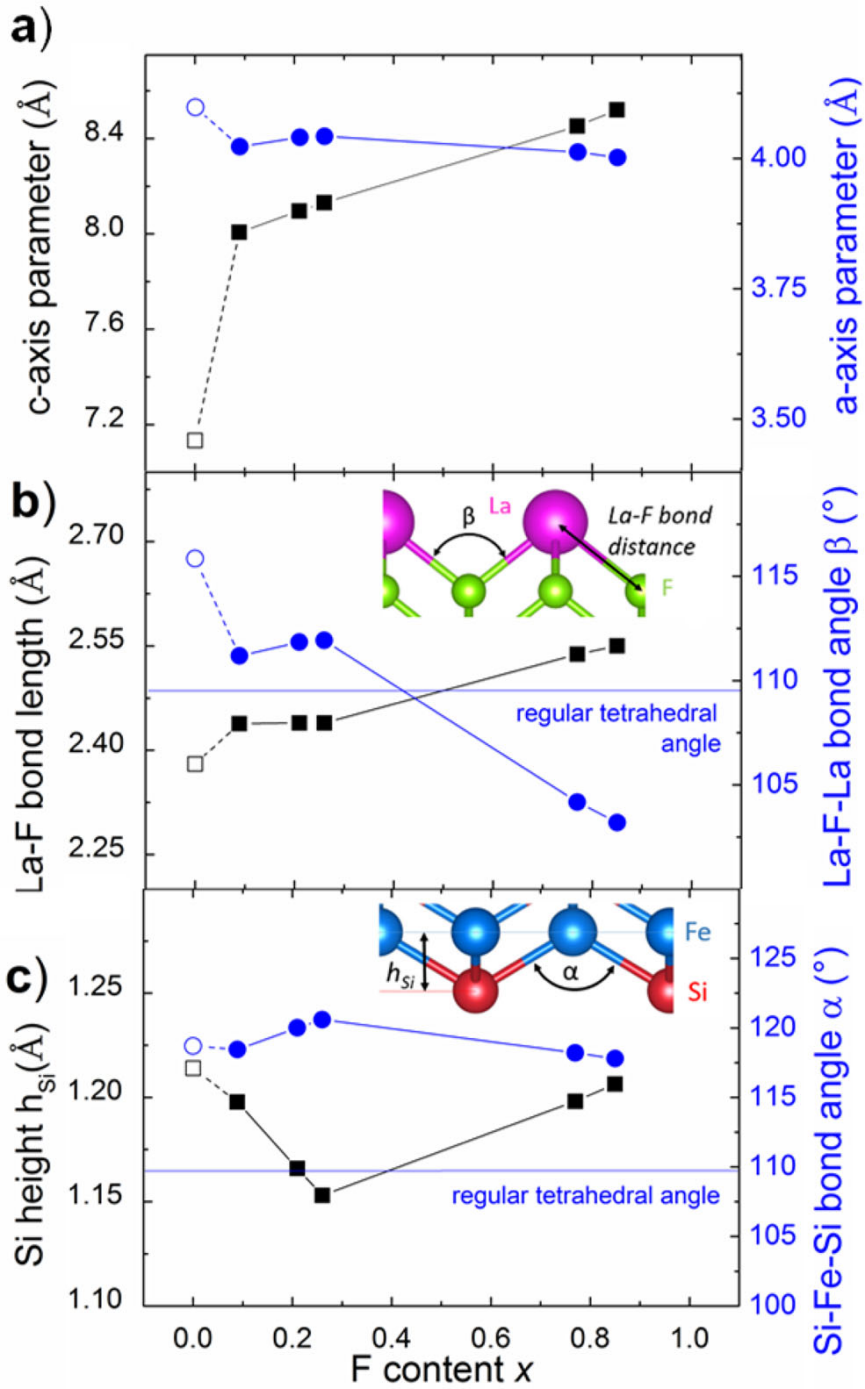
Structural parameters evolution upon fluorine insertion. **a** Evolution of the a-axis and c-axis parameters versus fluorine site occupancy, from single crystals refinements. The parent compound LaFeSi is also included. Although the fully occupied sample LaFeSiF was not directly obtained, we estimate its c-parameter at 8.64 Å by extrapolation. **b** Evolution of the La-F bond distance and the La-F-La bond angle, as a function of fluorine content (single crystals X-ray diffraction data). **c** F content dependence of the anion height h_Si_ above the Fe plane and of the Si-Fe-Si tetrahedral angle α. In the insets of **b** and **c**, the definition of the h_Si_ and angle parameters discussed in the **b** and **c** graphs are given.

We determined the LaFeSiF_*x*_ crystal structure from the XRD data. The F-site occupancy, in particular, depends on the growth conditions and was accurately determined from single-crystal XRD refinements for selected samples (see Supplementary Tables 1 and 2). Although refining the occupancy of a partially occupied F site may seem unaccurate, the analysis of the difference-Fourier maps after refinement without fluorine shows clearly a peak in the 2*b* Wyckoff position even for *x* = 0.09. The strong localization of the density may explain why we successfully refined the occupancy factor of fluorine position for low content values. Figure 4a shows the dependence of the c-axis parameter to the fluorine content. Strikingly, a fluorine intercalation as low as 9% produces a jump in the c-axis parameter with a + 12% variation —i.e. the same c-parameter variation of LaFeSi vs LaFeSiH. The c parameter then further increases linearly as a function of the F content x. The possibility of having non-stoichiometric compositions as well as the enhanced c-axis variation both result from electrostatic and steric effects of fluoride ion. On one hand, the hard fluoride ions act as strong pillars such that only few of them are necessary to stabilize the crystals (in contrast to the softer H ions, for example, which seem to require full occupancy^6,18^). On the other hand, as a consequence of the inductive effect, the more the ionic character of the LaF blocks, the more the covalency of the FeSi brick, leading to move away the two slabs. In that sense, the increase in the c-axis parameter is mostly governed by the F^-^ content through the charge transfer between the two groups of layers.

Figure 4a also shows a-axis parameter as a function of *x*, whose relative variation is much weaker compared to c. The La-F distance, in its turn, clearly reveals two different behaviors above and below *x* ~ 0.26 (see Fig. 4b). These two regimes can also be noticed in the rest of structural parameters, including a. These variations can be ascribed to the non-monotonic deformations of the La tetrahedron induced by F intercalation and are driven mostly by steric constraints. In LaFeSi, the distance between La atoms and the center of the tetrahedron is roughly 2.39 Å and therefore F anions can barely be accommodated without enlarging the cell (the lowest distance in LaF_3_ is 2.44 Å). At low F content, there is a first regime with a nearly constant bond length (2.44 Å), followed by a higher F content regime, where the La-F bond length linearly increases. This can be understood when looking at the deformation of the tetrahedron along the F concentration increase. Supplementary Fig. 14 shows the two tetrahedral angles La-F-La between La atoms belonging to the same tetrahedron. The La_4_ tetrahedra centered around F atoms tends to very slightly elongate in the a-axis direction at low fluorine concentrations. When more fluorine is intercalated, the tetrahedra distortion decreases in-plane and increases along the c-axis. Overall, the cell parameters evolution seems to depend on the balance between charge transfers involving mainly FeSi layers as well as La-Si covalent bonds (through inductive effects previously illustrated by XPS analysis) and steric effects induced by the low compressibility of the electronic cloud of the fluoride anion.

The height of the Si anion relative to the Fe plane h_Si_ and the corresponding tetrahedral angle α_Si-Fe-Si_ are shown in Fig. 4c. These parameters have been much discussed in relation to the superconducting properties of the FeAs-based systems^19^, providing optimal empirical values around 1.38 Å and 109° for h_As_ and α_As-Fe-As_ respectively. In LaFeSiF_*x*_ these values are similar to those of the analogous superconducting LaFeSiH (1.20 Å and 118.2°), but away from the optimal values found in arsenides. Once more, the inductive effects may play an important role regarding this phenomenon, combined with the differences in electronic polarizability between the Si and As atoms. Through inductive effects, the covalency of the FeSi sheet is then reinforced in LaFeSiF compared to LaFeSi. However, despite the La-F bond being more ionic than La-O, the inductive effect does not allow altering sufficiently the FeSi layer covalency compared to the Fe-As bond in LaFeAsO. The latter one therefore remains more covalent than Fe-Si because of the lower polarizability of Si and leads to a FeSi sheet slightly more shrunk along the c-axis, and eventually to a smaller h_Si_.

### Electrical and magnetic measurements and superconductivity across the LaFeSiF_*x*_ series

**Fig. 5:**
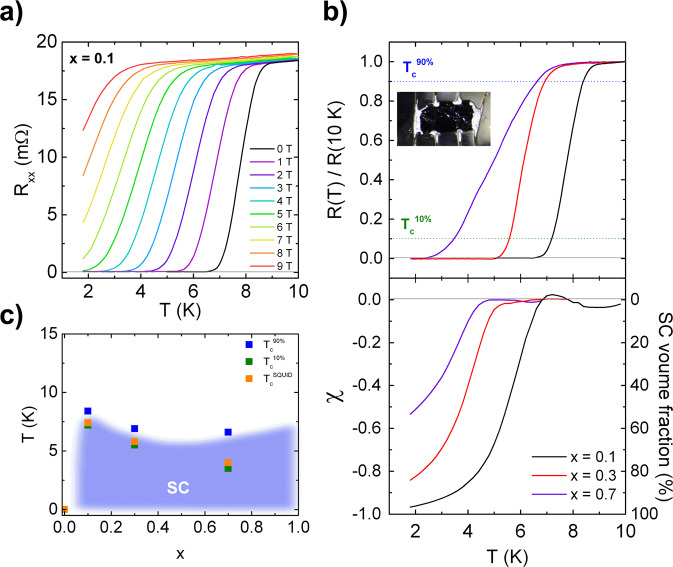
Transport and magnetic measurements. **a** Temperature dependence of the electrical resistance R of the LaFeSiF_0.10_ crystal at magnetic fields ranging from 0 to 9T. **b** Upper panel: temperature dependence of the electrical resistance of the LaFeSiF_*x*_ single crystals at *x* = 0.10, 0.30, and 0.70 normalized with respect to the resistance value measured at 10 K, with a picture of the LaFeSiF_0.3_ crystal in the inset. Blue and green dotted lines represent the criteria of 90% and 10% of the normal state résistance value used to defined T_c_^90%^ and T_c_^10%^, respectively. Lower panel: magnetic susceptibility of the same crystals measured in zero field cooled (ZFC) conditions at 0.5 mT, with field applied along the c-axis of the crystal. The signal was corrected from demagnetizing field. **c** Temperature–composition phase diagram summarizing the superconducting transition temperatures as measured by resistivity and magnetic susceptibility.

Figure 5 shows the electric and magnetic properties measured across the LaFeSiF_*x*_ series. Data are gained from single-crystals that are bigger than the ones used for the structural characterization above (see Methods). Figure 5a shows the in-plane electrical resistance measured in a LaFeSiF_0.10_ single crystal as a function of the temperature for different values of the magnetic field µ_0_H || c. At 0 T, superconductivity is observed with onset *T*_c_ at 9 K. The resistance drop shifts rigidly to lower temperatures as µ_0_H increases. We also found superconductivity in *x* = 0.30 and 0.70 single crystals with slightly lower onset *T*_c_ and a broader transition in the latter case (Fig. 5b, upper panel). We may attribute the broadening of the transition up to *x* = 0.7 to some spatial inhomogeneity of fluorine content within the sample.

In addition, the Hall coefficient R_H_ along the LaFeSiF_x_ series (*x* = 0, *x* = 0.1, and *x* = 0.3) has been measured (Supplementary Fig. 18) with the aim to extract the dominant type of carriers and the effective doping level. The values of *R*_H_ are positive for all samples at low temperature, indicating that charge transport is dominated by hole-like carriers in the range where superconductivity appears, in agreement with the values predicted by band structure calculation (see Supplementary Notes 4 in the Supplementary Information). The magnitude of *R*_H_ (10^−10^ m^3^/C) is at least one order of magnitude smaller than what is usually reported in the iron-based superconductors (IBSC) and both the *x* = 0.1 and *x* = 0.3 fluorides harbor a strong and non-trivial temperature dependence, with a sign change of *R*_H_ occurring at 35 K (20 K) for *x* = 0.1 (*x* = 0.3). Among IBSC, these characteristics are shared by the non-magnetic FeSe superconductor, where a compensated two-band model (i.e. *n*_h_ = *n*_e_) attributes the temperature dependence of *R*_H_ to a temperature dependence of the mobility of each type of carriers^20^. We therefore conclude that the LaFeSiF_*x*_ series is best described as a compensated metal, where orbital differentiation may drive the transport properties, as proposed for the multiband Hund’s metal Sr_2_RuO_4_^21^.

The lower panel of Fig. 5b shows the measured magnetic susceptibility which approaches −1, as expected from the Meissner effect (perfect diamagnetism) in the case of LaFeSiF_0.1_. While the superconducting volume fraction reaches 97% in the case of LaFeSiF_0.1_, it significantly decreases for higher doping level (85% and 53% volume fraction for *x* = 0.30 and 0.70 respectively). In any case, this confirms the bulk nature of the superconductivity observed in the LaFeSiF_*x*_ series (combined to the measurement of the heat capacity in Supplementary Figs. 19 and 20 and Supplementary Notes 5), with an upper critical field H_c2_ estimated as 11.5 T for LaFeSiF_0.1_ (Supplementary Fig. 22). Figure 5c displays the temperature–composition phase diagram, evidencing an extended region of superconductivity, from *x* = 0.1 to *x* = 0.7. Note that, even if the *T*_c_ is comparatively low, the robustness of such a superconducting state with respect to this type of changes in chemical composition is rather unique in the family of Fe-based superconductors.

### Electronic structure upon fluorine intercalation

The insertion of F into the spacer produces an abrupt increase in the c parameter that enhances the layered character of the crystal. This structural change towards a more 2D lattice can be expected to be amplified at the electronic level due to the inductive effect. In fact, by further increasing the ionic character of the LaF_*x*_ spacer towards the *x* = 1 limit, the electronic conduction will increasingly be due to the FeSi layer in a likely 2D fashion.

**Fig. 6:**
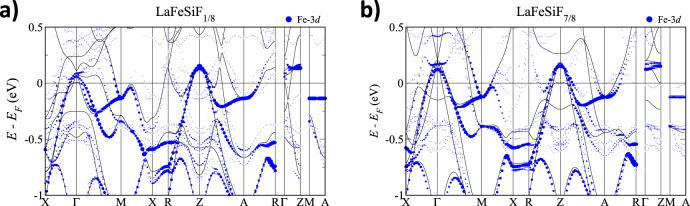
Electronic structure. Electronic band structure of LaFeSiF_*x*_ along a high symmetry path in the Brillouin zone with the Fe-3*d* character indicated in blue. The symbols correspond to direct supercell calculations for *x* = 1/8 (**a**) and *x* = 7/8 (**b**) with the structural parameters of the *x* = 0.09 and *x* = 0.85 crystals respectively. The lines indicate the bands of the LaFeSi precursor and of the LaFeSiF end-member, computed also with the same *x* = 0.09 and *x* = 0.85 single-crystal parameters respectively. The difference between these plots reveals non-stoichiometric effects; in particular, low-energy defect-like states associated to the LaF_*x*_ spacer and consolidated vs incipient portions of the Fe-3*d* Fermi surface around M (also related to the specific properties of the F atom).

Figure 6 shows the electronic structure computed for lightly (a) and heavily (b) fluorinated members of the LaFeSiF_*x*_ series. The trend obtained in these calculations confirms an increasingly 2D low-energy physics that becomes dominated by the Fe-3*d* states as expected from the inductive effect upon fluorination (see Supplementary Figs. 23 and 24 with a comparison with LaFeSi). For the heavily fluorinated compound, the comparison with direct supercell calculations shows that its electronic structure is essentially captured by that of LaFeSiF assuming the lattice parameters and internal atomic positions of the non-stoichiometric system. Its overall Fermi surface (FS) is indeed remarkably 2D, and much better resembles that of the reference pnictide LaFeAsO compared to the superconducting hydride LaFeSiH^18,22,23^ (see Supplementary Fig. 24). Thus, according to the fermiology that emerges from these results, superconductivity across this series can be readily interpreted in terms of the general picture widely accepted for the Fe-based superconductors (see e.g. Hirschfeld et al.^24^). The nesting between electron and hole FSs, however, is somewhat reduced not only because of their relative size but also due to the in-plane anisotropy of the former and non-stoichiometry effects. This is mostly detrimental for the *s* ± -wave pairing, yet the *d*-wave instability inherent to the electron FSs alone can be expected to survive^23^. At the same time, we note that the superconducting *T*_c_ increases by reducing the F content while the favorable features for superconductivity are gradually washed out, and completely disappear in the non-superconducting precursor LaFeSi. This suggests a non-trivial effect of electronic correlations across the LaFeSiF_*x*_ series, which may be stronger in low F limit. In fact, according to the inductive effect, the itineracy of the carriers within the FeSi layer can be expected to decrease with reducing the F content (in other words, metallicity in FeSi is enhanced when increasing the ionicity in the LaF_*x*_ layer). It then increases abruptly again in the non-superconducting LaFeSi precursor due its collapsed structure along c. Thus, the inductive effect, through the tuning of the La-F bond character, may provide an effective handle to control these correlations and thereby the functional properties of the system. In this respect, we note that LaFeSiF_0.1_ and LaFeSiH have similar T_c_’s. Following the above reasoning, this suggests a very close behavior in terms of charge transfer and ionicity, even if the hydride has full occupancy while the fluoride 10% only.

Note again that LaFeSiF_*x*_ is related, and at the same time fundamentally different from other Fe-based superconductors such as LaFeAs(O,F) when it comes to its synthesis. Specifically, LaFeSiF_*x*_ has been obtained from the LaFeSi precursor by means of the topochemical method that we demonstrate here. Thus, by means of topochemistry, we “functionalize” the precursor and could theoretically obtain parent compounds for stoichiometric amounts of F (e.g. *x* = 1). In LaFeAs(O,F), on the contrary, the ternary LaFeAs precursor simply does not exist, and F merely substitutes O in LaFeAsO (i.e. an already known material). Directly fluorinating an intermetallic precursor differs from the typical fluorination of ionic materials. To avoid decomposition, the fluorination reaction needs considerably less oxidizing conditions, which are very difficult to achieve with conventional methods.

In summary, we have introduced a topochemical fluorination route and demonstrated its potential for obtaining quantum materials with the synthesis of the LaFeSiF_*x*_ series. This series introduces an example of a topotactic fluorinated intermetallic compound, containing both metallic and ionic layers. Such a direct fluorine intercalation into metallic hosting structures has been remarkably elusive so far due to chemical decomposition issues. Overcoming this limitation has been possible via commercially available octacyclofluorobutane C_4_F_8_ and polytetrafluoroethylene. Other perfluorocarbon gases may be equally or even more effective as reactants, by playing with the covalent character of the C–F bond, which calls for further investigation. In addition, we have shown that the intercalation of fluoride anions into the structure has strong effects both on the structure and on the covalency of the FeSi layer via inductive effects. Such effects may be at the root of promoting superconductivity from LaFeSi with a F content as tiny as 9% and could explain the evolution of the properties for higher F content. Beyond this particular intermetallic, our work suggests a possible design rule to “activate” the latent functional properties of many other materials, which may span superconductivity, magnetism, mixed ionic-electronic conductivity for fluorine battery electrodes or sensors, or enhanced catalytic properties.

## Methods

### Synthesis

In a first step, polycrystalline LaFeSi samples, of masses between 2 and 4 g, were synthesized by arc melting in a high-purity argon atmosphere, from a stoichiometric mixture of pure elements with a La excess of 2% (Fe Alfa Aesar 99.95%, Si Alfa Aesar 99.9995%, La 99.9% Alfa Aesar). The boules were turned upside down and remelted several times to ensure chemical homogeneity. Prior to this step, pure La was pre-melted in an induction furnace for several minutes to evaporate volatile oxides and impurities. These samples were subsequently ground and cold pressed into pellets then annealed in evacuated quartz tubes for 10 days at 950 °C.

Hand-ground powders were then placed in a Pt boat inside a tubular Pt furnace and heated by induction to 500 °C for 1–4 h in a 1 bar static atmosphere of (C_4_F_8_ standard, Air liquide) or in the same atmosphere with a C_4_F_8_ flow of 10 mL.min^−1^ (details of the set-up are given in the Supplementary Fig. 25 of the supplementary materials). The obtained black powders were further ground, pelletized and annealed for 10 more days at 500 °C in vacuum to promote diffusion of the fluorine atoms and ensure their homogenization within the La-La layers. Cell parameters of the final compound strongly depend on the synthesis conditions: especially on the treatment duration, and on the nature of static or dynamic atmosphere.

Plate-like single crystals of LaFeSi were grown from natural cooling of an off-stoichiometric melt of composition La_35_Fe_35_Si_30_ after arc melting. As-grown crystals with size ranging from 300 × 300 µm^2^ to 1 × 1.5 mm^2^ were then subjected to the same fluorination and annealing steps described above for powders, resulting in 20–30-µm-thick brittle blackish platelet-like crystals.

In addition, fluorination treatment of the powders with polytetrafluoroethylene (PTFE, Sigma Aldrich) was carried out by mixing the appropriate amount of PTFE and base LaFeSi powders in a mortar, the mixture was sealed in a Pt tube in Ar atmosphere and treated at 400–700 °C for 12–72 h.

### X-ray diffraction

#### Powder

X-ray powder diffraction patterns were acquired using a PANalytical X’Pert Pro diffractometer (Cu-Kα radiation). The patterns were scanned over the 2θ angular range 8–100°. For each sample, we performed Rietveld refinements (with the Fullprof software^25^) to extract the structural parameters.

#### Single crystals

On the basis of their shape, several small (<40 × 40 × 10 µm^3^) single crystals were selected in the fluorinated samples upon the breaking of larger crystals. Data collection was performed using a Bruker Kappa Apex II diffractometer (Mo radiation) at room temperature. After the cell orientation-matrix searching procedure, data collection was performed at room temperature on the single crystals with the best quality of the intensity spots. Numerical absorption correction using the shape of the crystal (face indexed with the help of the video microscope) was made with SADABS-2014/5 software. All refinements were performed with the Jana2006 program package^26^. The structure was refined with the space group *P*4/*nmm* starting from the positions of the pristine compound LaFeSiH with F replacing H at the 0.25, 0.75, 0.5 position. Details of data collections and structure refinement can be found in the crystallographic information file (CIF) deposited in the Cambridge Crystallographic Data Centre with Deposition Number CSD 2051682-2051686. All structural parameters are given in Supplementary Tables 1 and 2. Fluorine content determination for the larger crystals was obtained from the linear relationship unveiled between the c-axis parameter and the F content when refining the small crystals.

### X-ray photoelectron spectroscopy

XPS surface analysis were performed using a Thermo-Fisher Scientific K-ALPHA spectrometer with a monochromatized Al Kα source (hν = 1486.6 eV) and a 200-μm X-Ray spot size. A pressure of 10−7 Pa was reached in the chamber when transferring the crystals. The full spectra (0−1100 eV) were obtained with a constant pass energy of 200 eV and high-resolution spectra (i.e. F1*s*, La3*d*, Fe2*p*, Si2 *s*) at a constant pass energy of 40 eV. Charge neutralization was applied during analysis and subsequent etching was achieved through low-energy Ar^+^ ions. High-resolution spectra were quantified using the AVANTAGE software provided by Thermo-Fisher Scientific (Scofield sensitivity factors applied).

### Transmission electron microscopy and electron diffraction

Electron diffraction experiments and the reconstruction of reciprocal space were carried out on a JEOL 2100 microscope, equipped with a double tilt specimen stage, operating at 200 kV. The powder was suspended in ethanol and a few drops of this was placed on a carbon-coated copper grid and air-dried before observation.

### Magnetic measurements

The magnetic susceptibility measurements were performed on single crystals with typical dimensions of 500 × 200 × 20 µm^3^ in Zero Field Cooled (ZFC) mode at 0.5mT using a Superconducting Quantum Interference Device (SQUID) magnetometer (MPMS XL, Quantum design). The single crystals used for the SQUID measurements are the very same samples as those used for the resistance measurements (including the quartz support and electrical gold leads, see below).

Additional measurements were performed on powdered samples with masses ranging between 10 and 20 mg. The samples (powder and single crystals) were placed in plastic capsules fixed inside a plastic straw (whose magnetization is negligible compared to that of the samples), at the appropriate position for centering with respect to the magnetic coils. For ZFC measurement, the sample is first cooled at zero magnetic field down to the lowest temperature 1.8 K. A magnetic field is then applied (along the c-axis in the cases of single crystals), and the magnetization of the sample is measured, either in a fixed magnetic field with increasing temperature (χ(T) measurements), or at a fixed temperature with increasing magnetic field (M(B) measurements). The magnetic susceptibility data have been corrected for the demagnetizing factor.

### Electrical measurements

The electrical transport properties were measured over a wide range of temperatures and magnetic fields up to 9T using a physical properties measurement system (PPMS, Quantum Design, using the resistivity option for compacted powders and the AC transport option for single crystals). Powder samples were compacted into 1 mm thick and 3 mm diameter pellets. Silver wires were contacted using silver paste (Dupond 4929) onto the sample surface in the aligned four-point probe configuration, and measured with a 1 mA current. Single crystals with typical dimensions of 500 × 200 × 20 µm^3^ were contacted using 20 µm gold wires and silver paste (Dupond 4929) to measure the sample resistance using a standard four points method. AC resistivity measurements were performed using currents ranging from 10 µA to 1 mA, at a frequencies ranging from 10 Hz to 300 Hz, for magnetic fields up to 9T applied along the c-axis of the crystals. The Hall coefficient *R*_H_ has been extracted by measuring the off-diagonal term of the resistivity tensor *ρ*_xy_ through field sweeps between −9T and +9T, with an excitation current up to 1 mA. The data have been anti-symmetrized to remove any contamination from *ρ*_xx_ due to a slight misalignment between the voltage contacts. The odd component has been extracted as *ρ*_xy_ = (*ρ* (+B) − *ρ* (−B))/2.

### Heat capacity measurements

Heat capacity measurements at zero magnetic field were performed using a standard relaxation technique, as implemented in our experimental setup (PPMS, Quantum Design). As the single crystals studied in this work were too small, with a typical mass of 30–100 µg, we have conducted heat capacity measurements on compacted powder sample, with a mass of 20–30 mg. The sample was glued to the sample holder using Apiezon N-grease and the contribution of the sample holder and grease was measured just before to subtract it to the total heat capacity measurement.

### Band structure calculations

The electronic-structure calculations were performed using the all-electron code WIEN2k^27^ based on the full-potential augmented plane-wave plus local orbitals method (APW + LO). The results (main text and Supplementary Information) were obtained in the PBE generalized gradient approximation^28^, using experimentally determined structural parameters. The local density approximation (LDA)^29^ was also used for comparison, which yielded essentially the same results. We choose muffin-tin radii of *R*_La_^MT^ = 2.50 a.u., *R*_Fe_^MT^ = 2.40 a.u., *R*_Si_^MT^ = 2.00 a.u., and *R*_F_^MT^ = 1.90 a.u. with the cutoff *R*_MT_.*K*_max_ = 7.0. The band structure calculations for the stoichiometric cases were performed using a tetragonal cell (*P*4/*nmm* symmetry) encompassing 2 formula units, a 16 × 16 × 8 k-mesh was employed for the self-consistent calculation. For the non-stoichiometric compositions, a 2 × 2 × 2 tetragonal supercell was used, with an 8 × 8 × 4 k-mesh giving an equivalent sampling to the one we used for the stoichiometric compound. The band structure of the supercell was unfolded into the Brillouin zone of the primitive cell using the unfolding algorithm as implemented in the Fold2Bloch post-processing tool^30,31^. The original Fold2Bloch was adapted to treat the unfolding of the character of the bands.

## Supplementary information

Supplementary Information

## Data Availability

Details of data collections and structure refinement can be found in the crystallographic information file (CIF) deposited in the Cambridge Crystallographic Data Centre with Deposition Number CSD 2051682-2051686. The authors declare that all other data supporting the findings of this study are available within the paper and its Supplementary Information file. Additional data related to this paper are available from the corresponding authors upon reasonable request.

## Code availability

WIEN2k code used for DFT calculations is available at http://www.wien2k.at/ and Jana2006 program package used for single-crystal XRD refinements is available at http://jana.fzu.cz.
