# Supplementary material for: Topotactic fluorination of intermetallics as an efficient route towards quantum materials
Source: Nat Commun. 2022 Mar 18;13:1462. doi: 10.1038/s41467-022-29043-8 (PMC8933527; doi:10.1038/s41467-022-29043-8)
Supplement: Supplementary file 1 — Supplementary Information [file 41467_2022_29043_MOESM1_ESM.pdf]

Supplementary Information

Topotactic fluorination of intermetallics as an efficient route towards quantum materials

Jean-Baptiste Vaney[1], Baptiste Vignolle[1], Alain Demourgues[1], Etienne Gaudin[1], Etienne Durand[1], Christine Labrugère[2], Fabio Bernardini[3], Andrés Cano[4], Sophie Tencé[1*]

[1] *CNRS, Université Bordeaux, Bordeaux INP, ICMCB, UMR 5026, Pessac, France*

[2] *CNRS, Univ. Bordeaux, PLACAMAT UMS 3626, Pessac, F-33600, France*

[3] *Dipartimento di Fisica, Università di Cagliari, IT-09042 Monserrato, Italy*

[4] *CNRS, Université Grenoble Alpes, Institut Néel, 38042 Grenoble, France*

* corresponding author : sophie.tence@icmcb.cnrs.fr

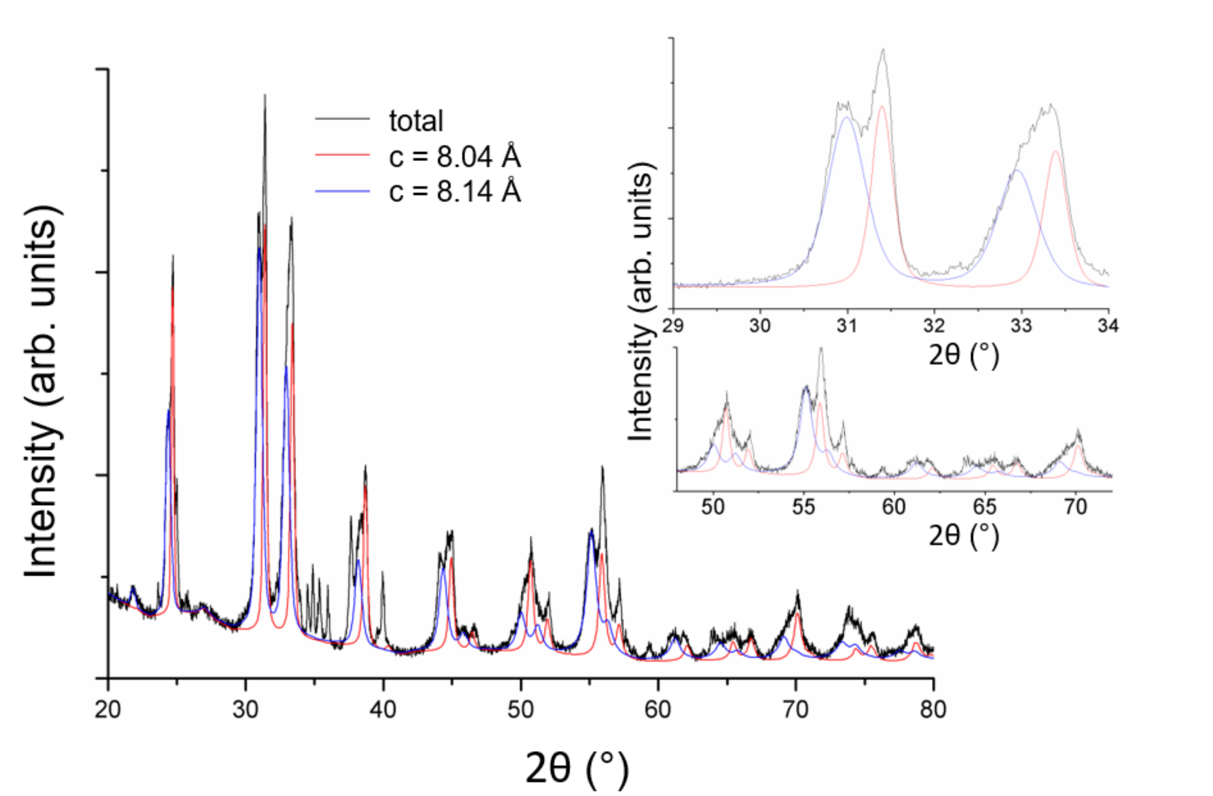

Supplementary Figure 1. XRD pattern of a LaFeSi sample fluorinated with C_4F_8 at 500°C for 2h. A phase separation into two distinct fluorinated phases in red and blue is observed. The insets present a zoom of the pattern around 2θ=32° and 2θ=60°.

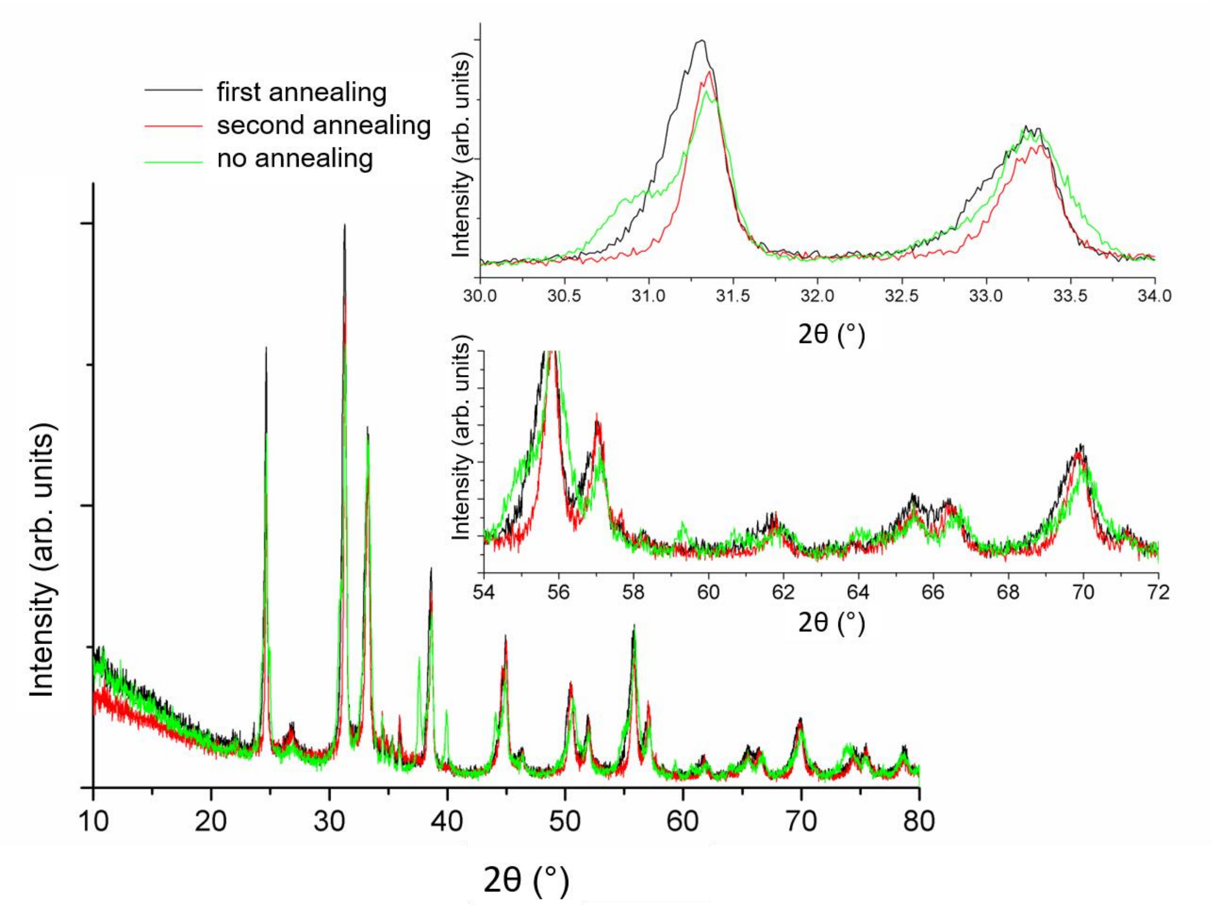

Supplementary Figure 2. XRD patterns of a LaFeSi sample fluorinated with C_4F_8 at 500°C for 2h and subject to two annealing steps of 5 days each. The progressive vanishing of the second fluorinated phase is observed. The insets present a zoom of the patterns around 2θ=32° and 2θ=63°.

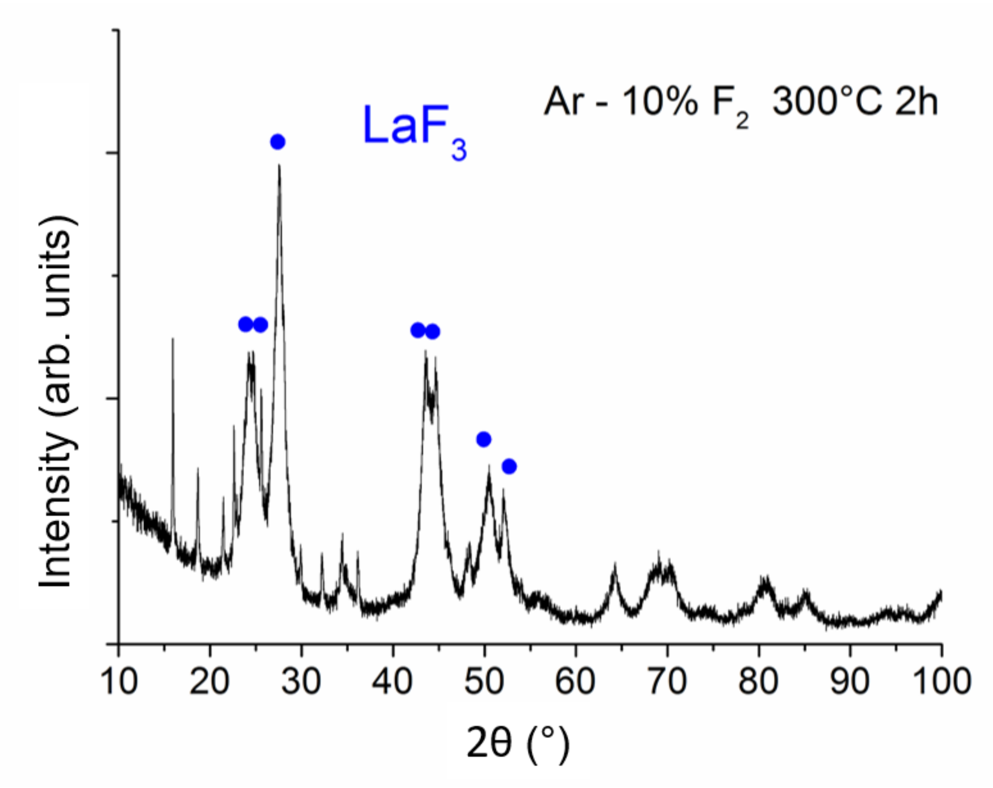

Supplementary Figure 3. XRD pattern of LaFeSi after F_2 gas treatment (Ar-10% F_2, 300°C, 2h).

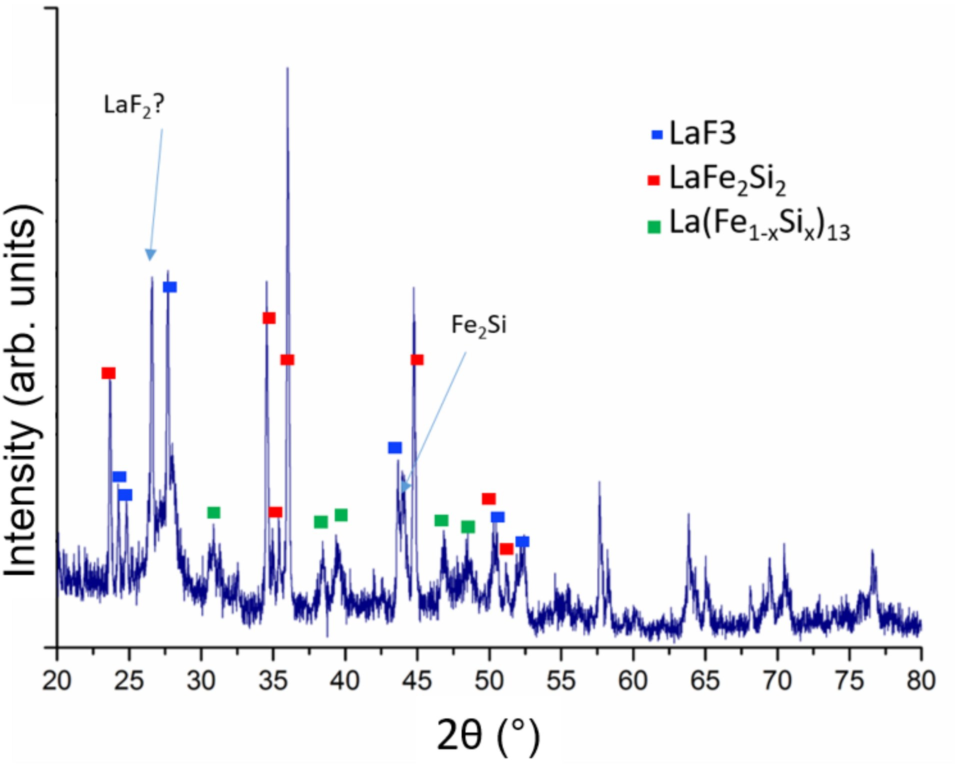

Supplementary Figure 4. XRD pattern obtained after solid-state route (LaFeSi+LaF_3+FeSi) at 950°C for 10 days.

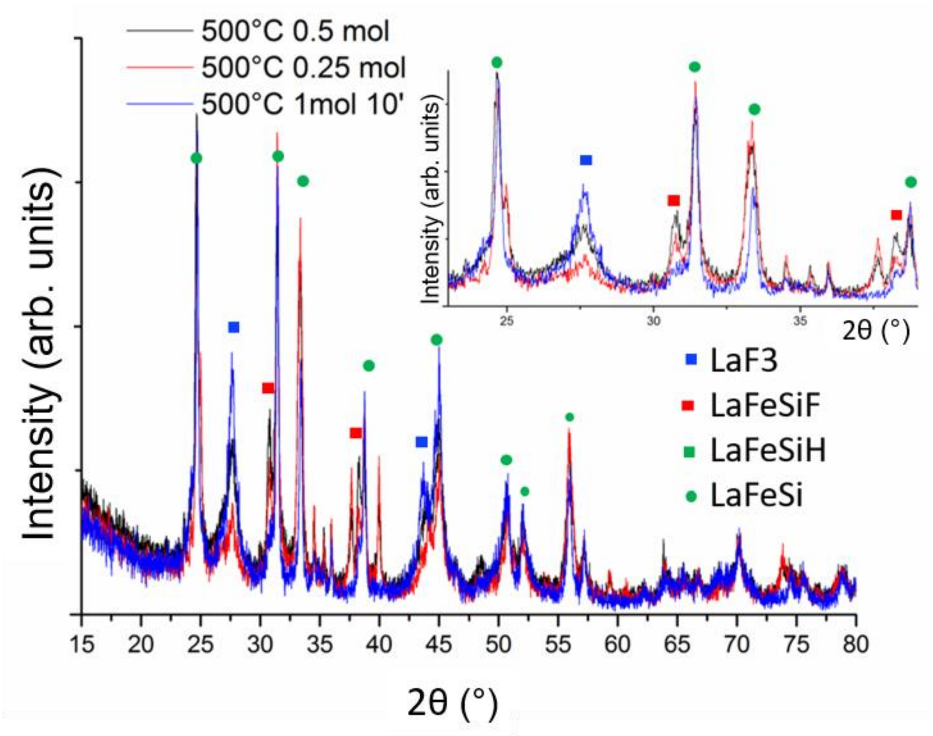

Supplementary Figure 5. XRD patterns after NH_4F treatment on LaFeSi at 500°C under different conditions. The insets shows a zoom of the patterns around 2θ=30°.

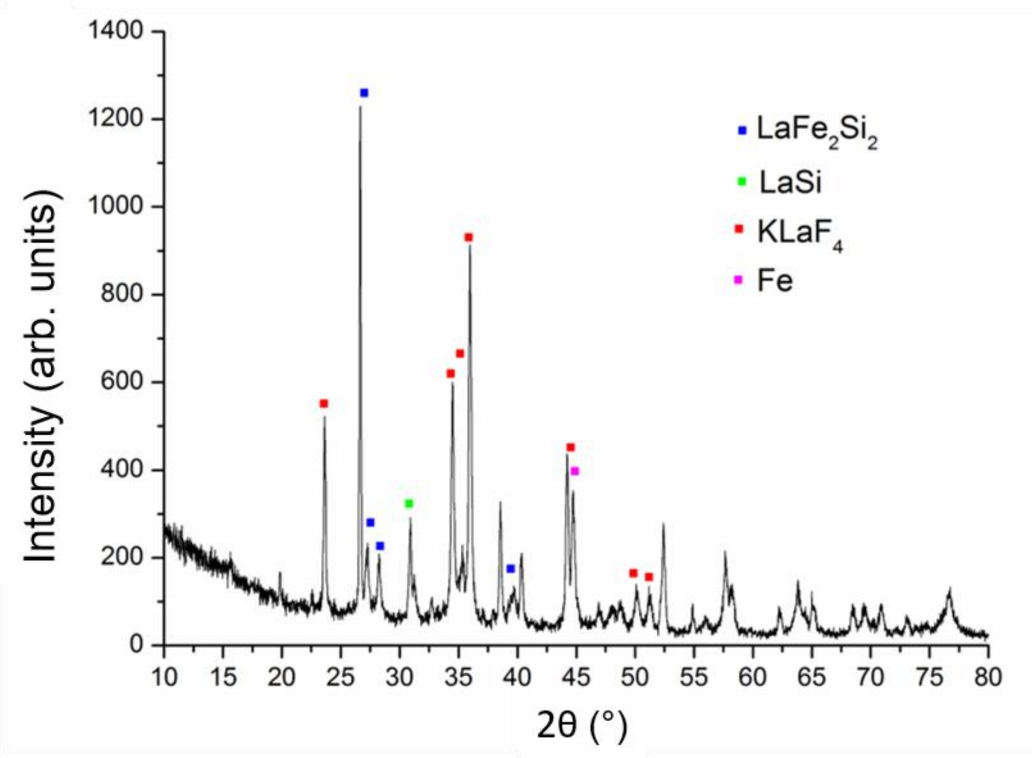

Supplementary Figure 6. XRD pattern obtained after KF treatment on LaFeSiH at 800°C.

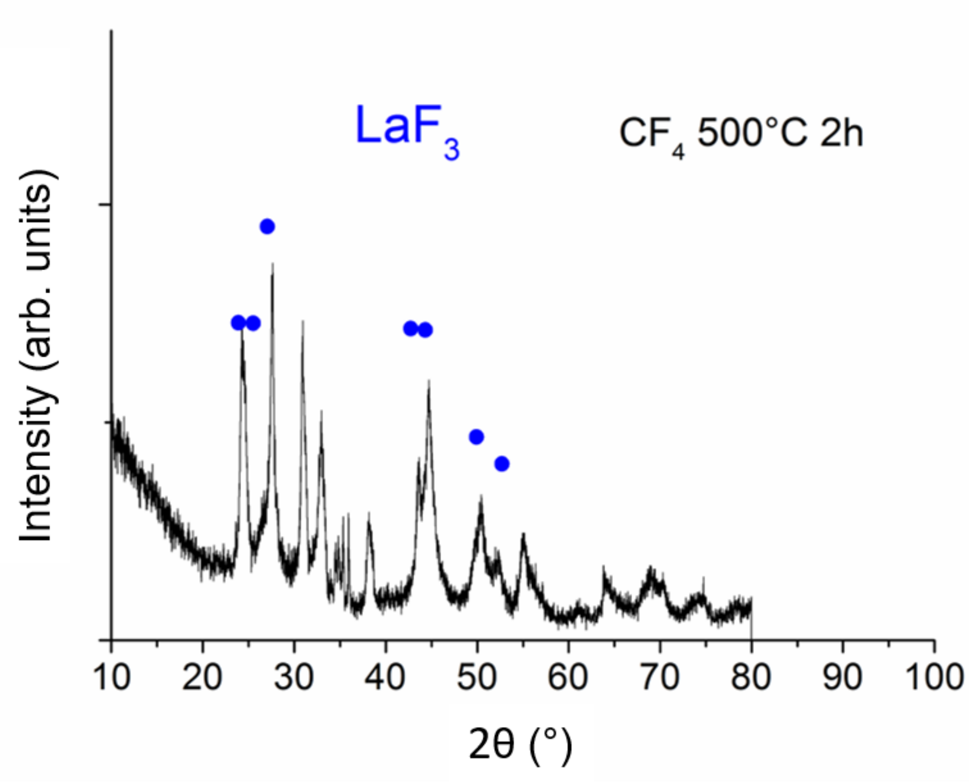

Supplementary Figure 7. XRD pattern obtained after CF_4 treatment on LaFeSi at 500°C for 2h.

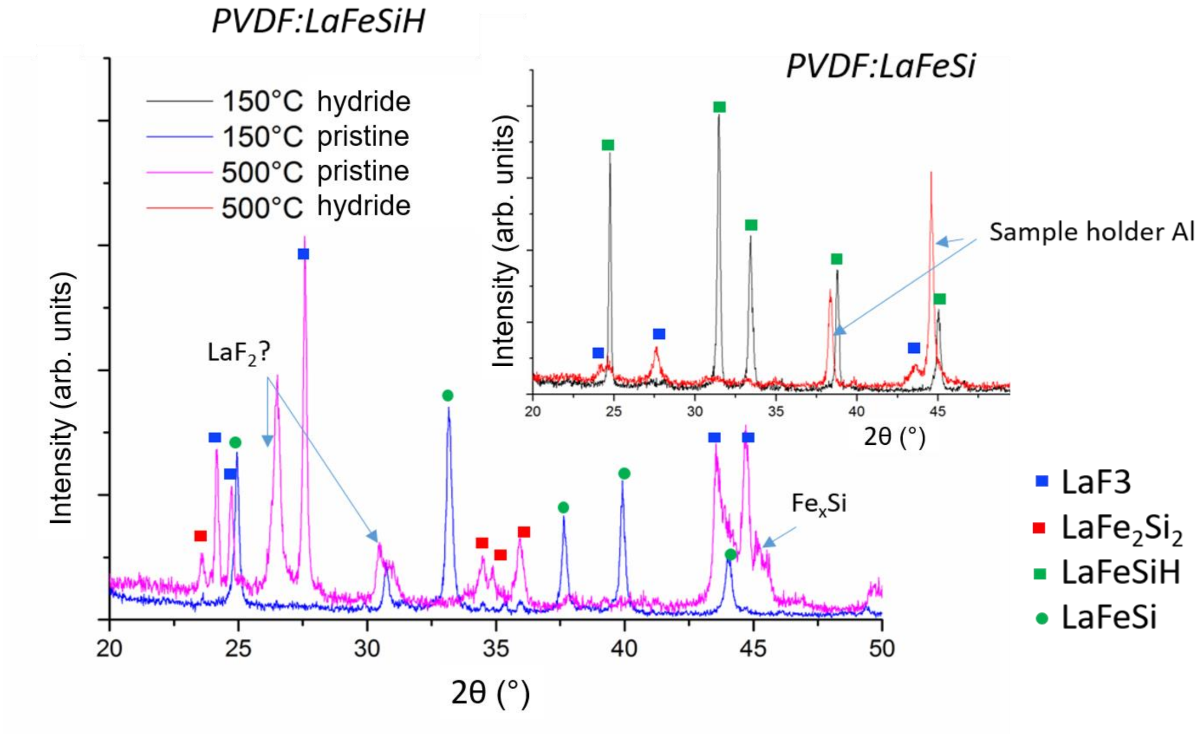

Supplementary Figure 8. XRD patterns obtained after PVDF treatment at different temperatures on LaFeSiH and LaFeSi (inset).

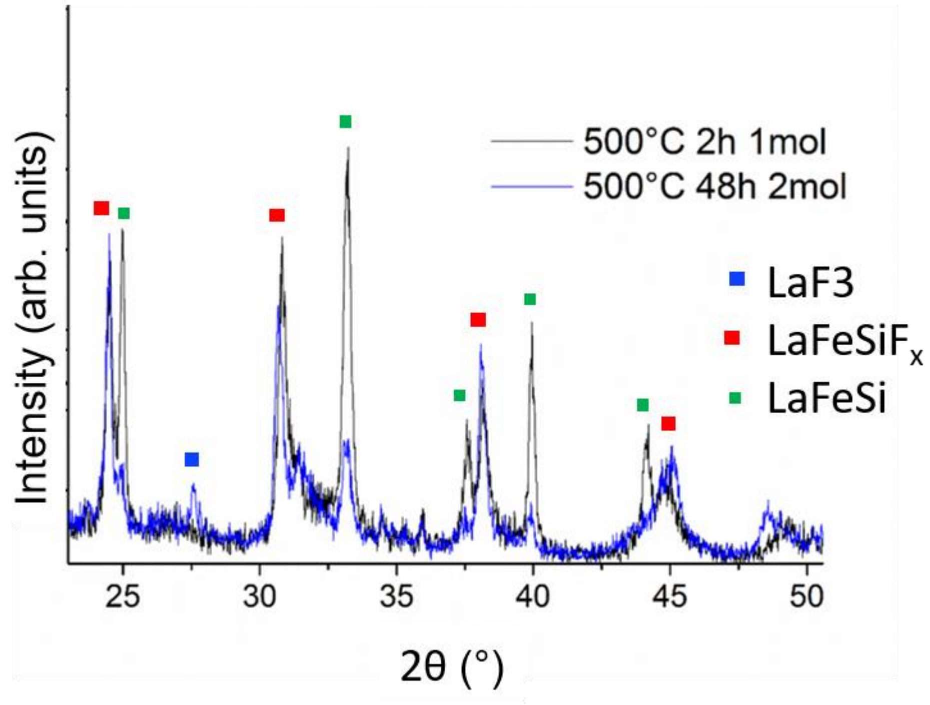

Supplementary Figure 9. XRD patterns obtained after PTFE treatment of LaFeSi at 500°C for two different procedures. Noteworthy, LaF_3 appears before all LaFeSi has reacted.

Supplementary Notes 1

X-Ray Photoelectron Spectroscopy: Three single crystals were measured in XPS: LaFeSi, $LaFeSiF_{0.1}$ and $LaFeSiF_{0.3}$. The obtained results are shown respectively in Supplementary Figures 10, 11 and 12. Upon etching of the surface, a signal more representative of the bulk can be obtained at La 3*d*, Fe 2*p*, Si 2*s* and F1*s* energy ranges. For LaFeSi, a peak in the binding energy range of fluorine is absent while it is noticeably present for both $LaFeSiF_{0.1}$ and $LaFeSiF_{0.3}$. The intensity of the F1*s* peaks scales with the fluorine content extracted from cell parameters, confirming the presence of F within the structure. In addition, for $LaFeSiF_{0.3}$, the larger peak width at the F1*s* binding energy hints at a less homogeneous distribution of F within the structure. The binding energy of the F1*s* electrons is located at 685 eV in these compounds, differing from the energies typical of LaF_3 and LaOF (respectively 687 eV[1] and 684 eV[2]), once again confirming the bulk character of the F insertion.

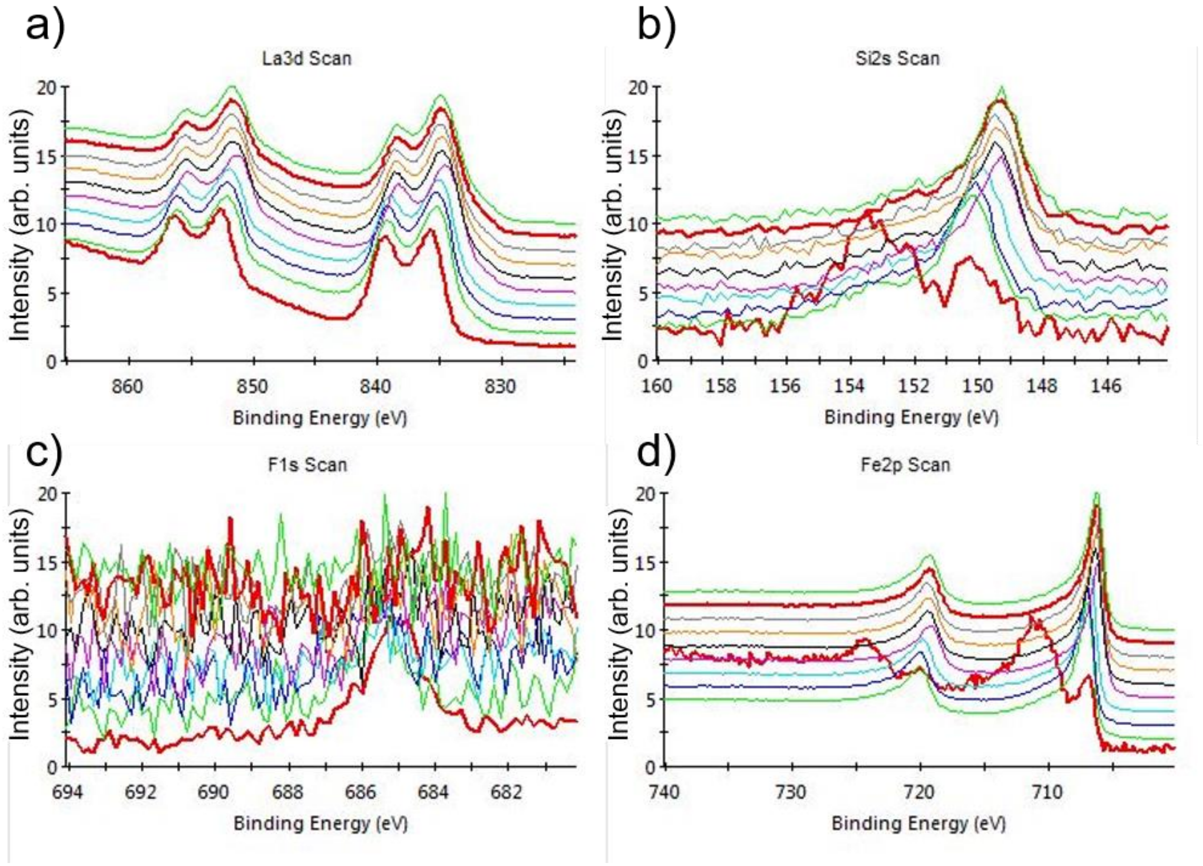

Supplementary Figure 10. XPS signal (La3*d* (a), Si2*s* (b), F1*s* (c), Fe2*p* (d)) on the surface for a LaFeSi single crystal. The lowest curve is the one at the very surface and each upper one was recorder after an etching cycle. The upper one therefore indicates the signal of the elements in the bulk. The occurrence of fluorine at the surface (before etching) of LaFeSi compound is due to pollution induced by etching of other fluoro-silicide samples during XPS analysis under secondary vacuum.

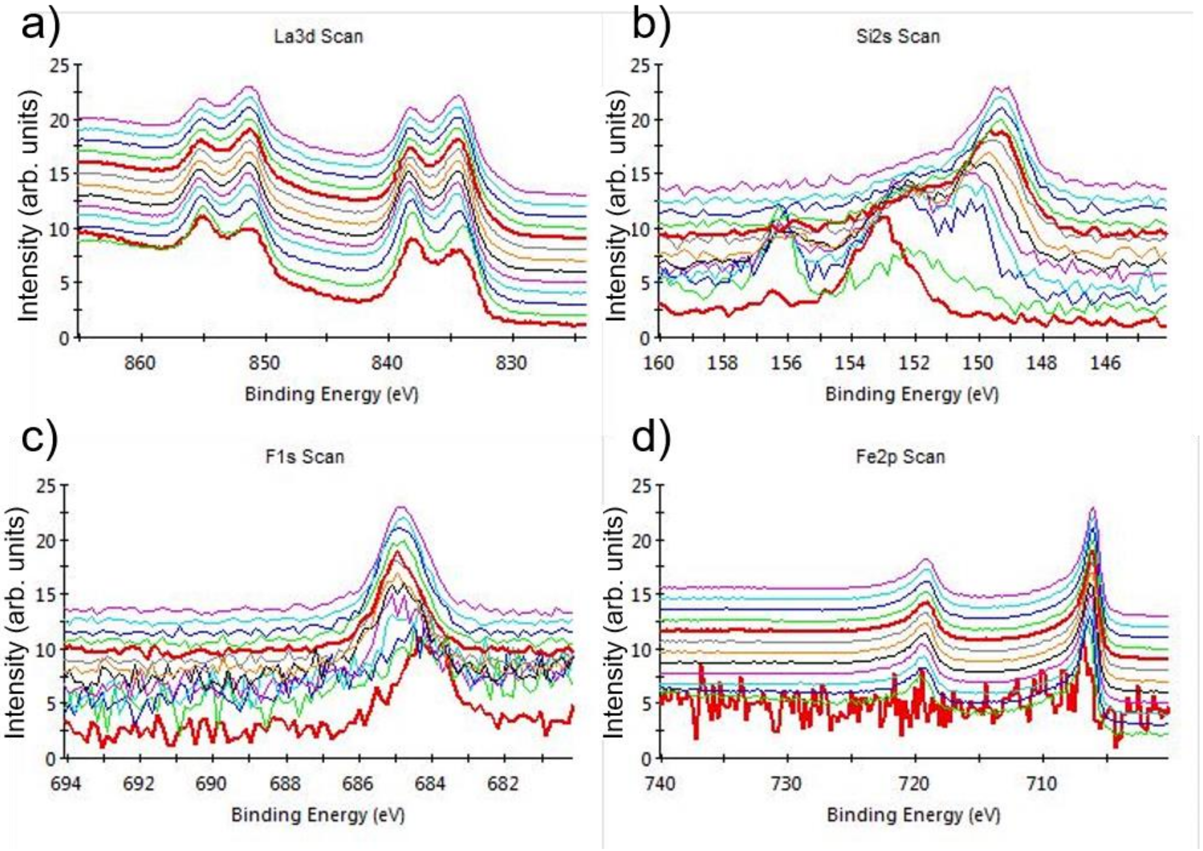

Supplementary Figure 11. XPS signal (La3*d* (a), Si2*s* (b), F1*s* (c), Fe2*p* (d)) on the surface for a $LaFeSiF_{0.1}$ single crystal. The lowest curve is the one at the very surface and each upper one was recorder after an etching cycle. The upper one therefore indicates the signal of the elements in the bulk. The presence of fluorine atoms is clearly evidenced compared to the case of LaFeSi.

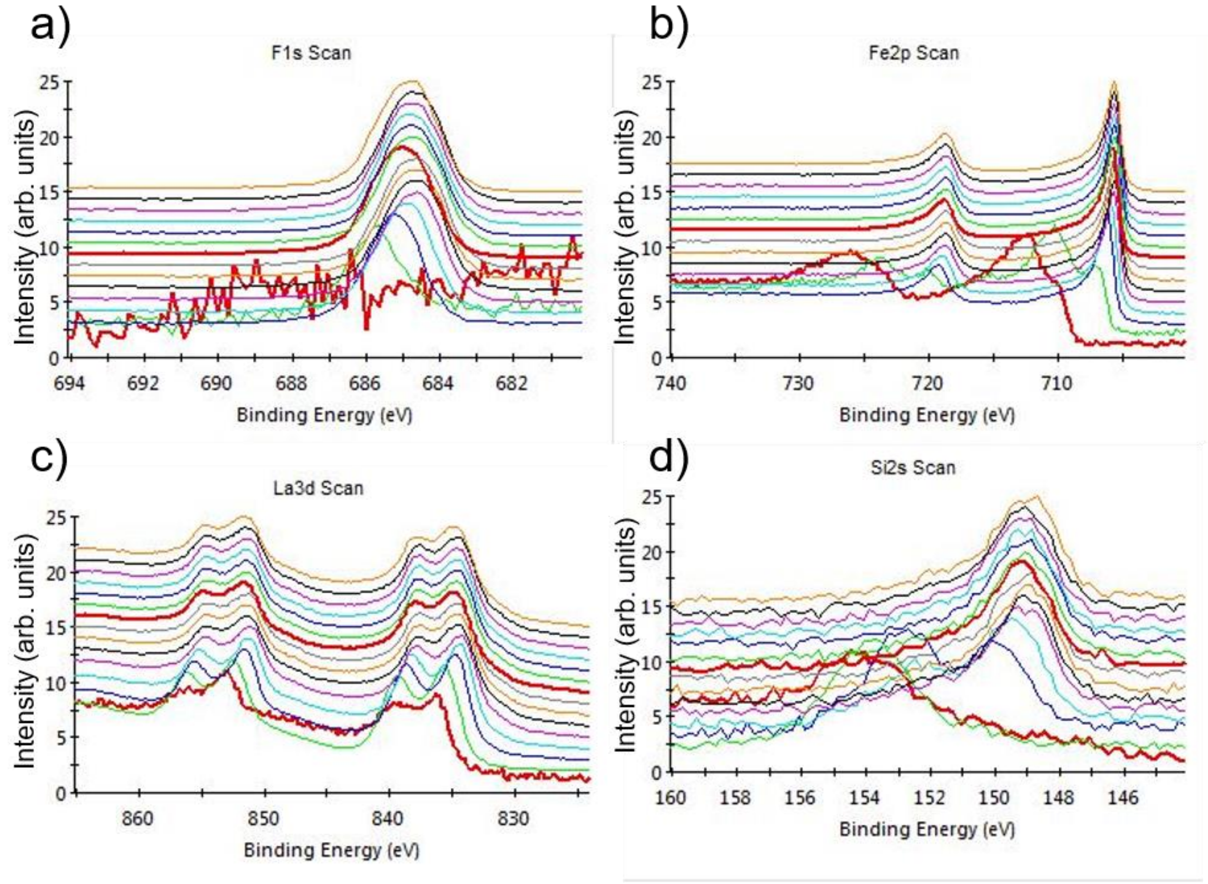

Supplementary Figure 12. XPS signal (F1*s* (a), Fe2*p* (b), La3*d* (c), Si2*s* (d)) on the surface for a $LaFeSiF_{0.3}$ single crystal. The lowest curve is the one at the very surface and each upper one was recorder after an etching cycle. The upper one therefore indicates the signal of the elements in the bulk. The presence of fluorine atoms is clearly evidenced compared to the case of LaFeSi.

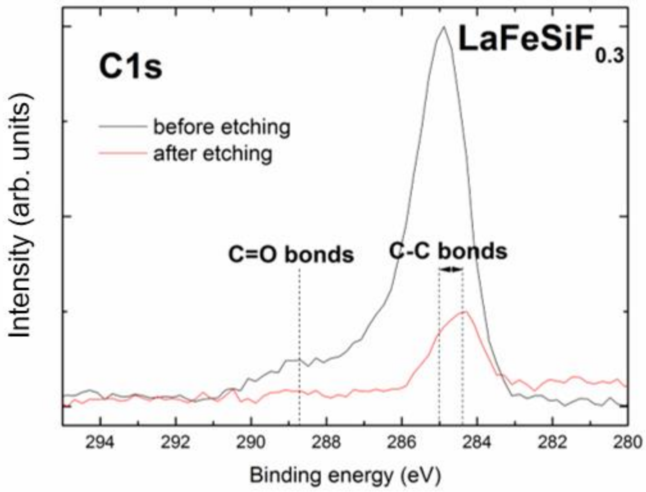

Supplementary Figure 13. XPS spectra at the C1*s* binding energy before (in black) and after etching at 70 nm (in red) of a $LaFeSiF_{0.3}$ single crystal. The energy range between 285 and 284.5 eV is typical of *sp*^{2}-like C-C bonds such as in graphene[3,4]. The absence of peaks in the range 283.5-282 eV confirms the absence of C-metal bonds.

The cell parameters, as well as the quality parameters of the refinements, obtained from refinement of the X-ray diffraction data of five different single crystals are given in Supplementary Table 1. The structural parameters obtained from these refinements are given in Supplementary Table 2.

Supplementary Table 1. Crystal data, Experimental details and Structure refinement

**Crystal data**					
Chemical formula	$LaFeSiF_{0.09}$	$LaFeSiF_{0.21}$	$LaFeSiF_{0.26}$	$LaFeSiF_{0.77}$	$LaFeSiF_{0.85}$
M_r (g.mol^{-1})	224.5	226.8	227.6	237.5	239.0
Crystal system, space group	Tetragonal, *P4/nmm* origin choice 2				
Temperature (K)	293				
a (Å)	4.0228(7)	4.0406(4)	4.0425(4)	4.0052(3)	3.9960(3)
c (Å)	8.007(3)	8.096(2)	8.131(2)	8.4527(13)	8.5158(14)
V (Å^3)	129.57(5)	132.19(3)	132.88(3)	135.60(3)	135.98(3)
Density (XRD, Z = 2	5.755	5.699	5.688	5.816	5.837
Radiation type	Mo K_α radiation, λ = 0.71073 Å				
μ (mm^{-1})	21.93	21.50	21.39	21.00	20.95
Crystal size (μm^3)	35×23×6	50×40×10	39×24×10	50×45×14	75×49×12
**Data collection**					
Diffractometer	Bruker CCD diffractometer APEX II				
Absorption correction	Numerical, SADABS				
T_{min}/T_{max}	0.506, 1.000	0.403, 0.861	0.539, 0.858	0.385, 0.754	0.235, 0.851
θ range (°)	2.5 – 31.7	2.5 – 32.0	2.5 – 30.3	2. 4 – 32.3	2. 4 – 32.0
Range for h, k, l	±5, ±5, ±11	-6/5, -6/5, ±12	±5, -4/5, ±11	-5/6, ±6, ±12	±5, ±5, ±12
No. of measured reflections	2989	4409	2023	5129	4411
No. of independent reflections	161	170	147	178	168
No. of reflections with $I > 2\sigma(I)$	141	147	112	175	160
R_{int}	0.065	0.063	0.085	0.042	0.045
**Refinement**					
$R[F^2 > 2\sigma(F^2)]$, $wR(F^2)$	0.023, 0.047	0.022, 0.052	0.028, 0.057	0.025, 0.056	0.021. 0.041
Goodness-of-fit S	1.16	1.27	1.09	1.54	1.03
No. of reflections	161	170	147	178	168
No. of parameters	11	11	11	12	12
No. of restraints	0	0	0	0	0
$\Delta\rho_{max}$, $\Delta\rho_{min}$ (e Å^{-3})	1.52, -1.00	1.94, -1.90	2.49, -2.22	2.42, -2.07	1.43, -1.03

Supplementary Table 2. Atomic positions and equivalent displacement parameters of $LaFeSiF_x$ for x = 0.09, 0.21, 0.26, 0.765, 0.854.

Position	Wyck.	*x*	*y*	*z*	*U*iso*/*U*eq (Å^2)	*Occ (<1)*
La1	2*c*	1/4	1/4	0.67205(8)	0.01084(15)	
Fe1	2a	3/4	1/4	0	0.0116(3)	
Si1	2*c*	1/4	1/4	0.1496(4)	0.0128(6)	
F1	2b	3/4	1/4	1/2	0.02(2)*	0.09(2)

Position	Wyck.	*x*	*y*	*z*	*U*iso*/*U*eq (Å^2)	*Occ (<1)*
La1	2*c*	1/4	1/4	0.66877(8)	0.01204(14)	
Fe1	2a	3/4	1/4	0	0.0129(3)	
Si1	2*c*	1/4	1/4	0.1440(4)	0.0155(6)	
F1	2b	3/4	1/4	1/2	0.018(7)*	0.21(2)

Position	Wyck.	*x*	*y*	*z*	*U*iso*/*U*eq (Å^2)	*Occ (<1)*
La1	2*c*	1/4	1/4	0.66790(13)	0.0119(2)	
Fe1	2a	3/4	1/4	0	0.0117(5)	
Si1	2*c*	1/4	1/4	0.1418(6)	0.0139(11)	
F1	2b	3/4	1/4	1/2	0.009(9)*	0.26(2)

Position	Wyck.	*x*	*y*	*z*	*U*eq (Å^2)	*Occ (<1)*
La1	2*c*	1/4	1/4	0.68451(6)	0.01075(13)	
Fe1	2a	3/4	1/4	0	0.0101(3)	
Si1	2*c*	1/4	1/4	0.1418(3)	0.0115(5)	
F1	2b	3/4	1/4	1/2	0.0075(13)	0.765(13)

Position	Wyck.	*x*	*y*	*z*	*U*eq (Å^2)	*Occ (<1)*
La1	2*c*	1/4	1/4	0.68597(5)	0.01050(11)	
Fe1	2a	3/4	1/4	0	0.0099(2)	
Si1	2*c*	1/4	1/4	0.1416(3)	0.0110(4)	
F1	2b	3/4	1/4	1/2	0.0084(11)	0.854(11)

Supplementary Notes 2

Deformation of the La_4 tetrahedron due to F insertion: By inductive effect, the more the ionic character of the LaF blocks, the more the covalency of the FeSi brick, leading to move away the two slabs. In that sense, the increase in *c* will be mostly governed by the F^- content through the charge transfer between the two groups of layers. At any content, the La-F bond has a lower boundary that should lie between 2.42-2.44 Å (these are the lowest distances in LaF_3). In LaFeSi, the distance between La atoms and the center of the tetrahedron is 2.39 Å and therefore no F atom can be accommodated without enlarging the cell. When a low amount of fluorine is introduced, the La-F bond distance thresholds around 2.44 Å up to at least x=0.26, hinting at this distance at the minimum possible distance of the La-F bond in this material. When increasing the F content, the *a*-axis parameter slowly increases before then decreasing for higher F content as shown in Figure 4 of the article body. This can be understood by the deformation of the tetrahedron along the F concentration increase. Supplementary Figure 14 shows the tetrahedral angle La-F-La between La atoms belonging to the same tetrahedron: for the same layer and a different layer. The La_4 centered around F atoms tend to very slightly elongate in-plane when the fluorine concentration is low enough, due to lack of available space in the out-plane direction, eventually forcing an cell expansion in the plane. When more fluorine is intercalated, hence increasing the hole concentration in the FeSi layer and allowing for a larger cell along the *c*-axis, the tetrahedra distortion decreases in-plane and increases along the out-of-plane direction. Overall, the cell parameters evolution depends on the balance between charge transfers between the layers and steric effects induced by the low compressibility of the electronic cloud of the fluoride anion.

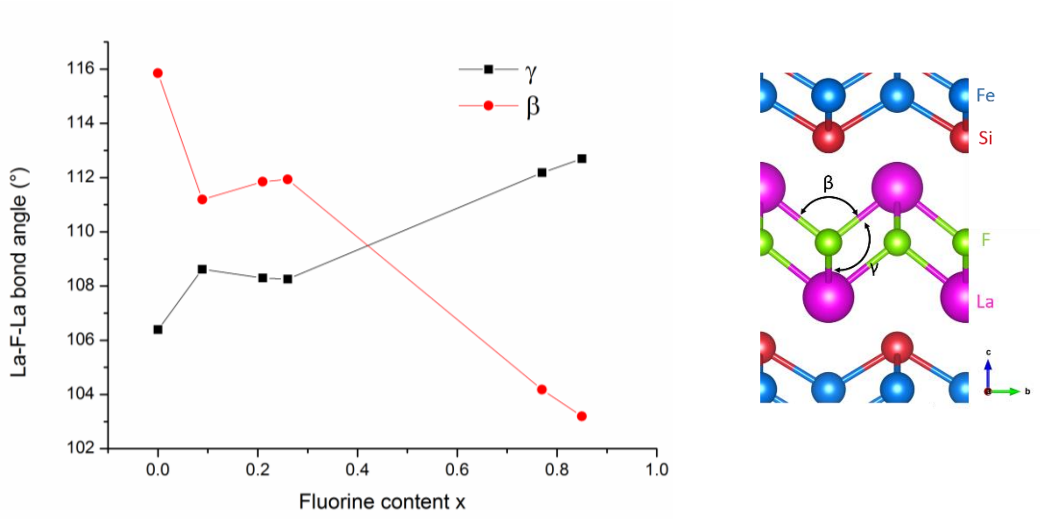

Supplementary Figure 14. Evolution of the angles γ and β in the La_4F tetrahedron along the F intercalation rate. The definition of γ and β is given on the picture of the structure (right).

Supplementary Notes 3

TEM investigation of $LaFeSiF_x$: F order/disorder: For low F content, when fluoride anions are scarce but in enough quantity to move the La layers away, the flatness of the La layer is locally perturbed. One evidence of this behavior is given by electron diffraction patterns of two different crystals of $LaFeSiF_x$ (x determined from *c* value), shown in Supplementary Figure 15 (a) (x=0.20) and Supplementary Figure 15 (b) (x=0.35). On Supplementary Figure 15 (a), for the lower F-content crystal, diffuse scattering (due to La atoms slightly off the layer) along the *c*-axis is visible while being absent in Supplementary Figure 15 (b) for the higher F-content crystal.

In addition, some discrepancy of the *a* parameter observed at fixed *c* hints at the possibility of complex arrangements of F atoms within the La layer, especially at rather low F rate. These slightly different *a*-parameters found for a unique *c* can be explained when homogeneous diffusion of F is not fully achieved upon annealing, probably due to the existence of several metastable phases with the same F content at the annealing temperature. At low *x*, F^- ions can arrange locally to avoid F^- first neighbors, unfavorable from an electrostatic point of view, allowing for a low value of *a*. But when distribution is not homogeneous and can be frozen from a more disordered high temperature state (by quenching for example), a different value of *a* can arise, with *c* unchanged.

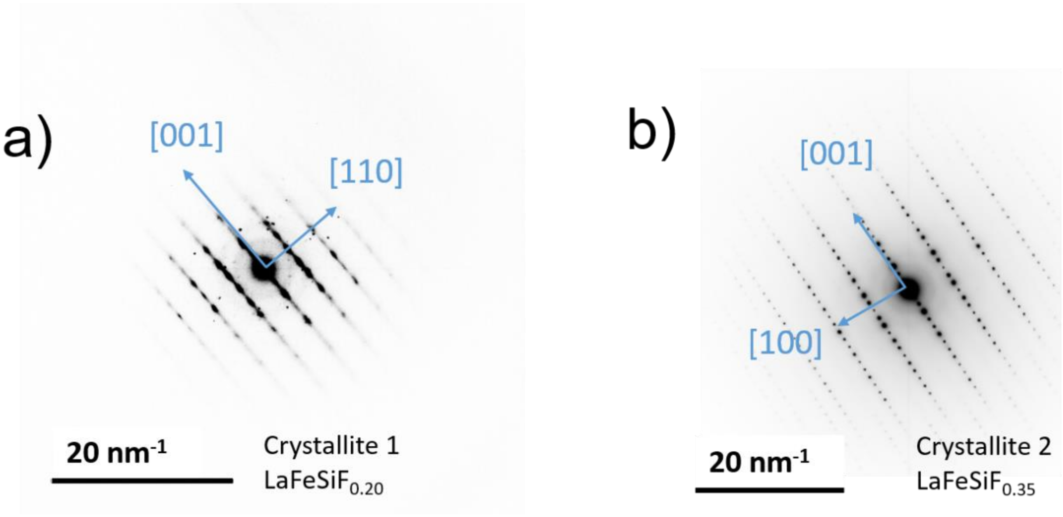

Supplementary Figure 15. Electron diffraction patterns of two crystallites with different F content (a and b). Diffusion along c-axis is prominent in the first pattern while it is absent in the second one.

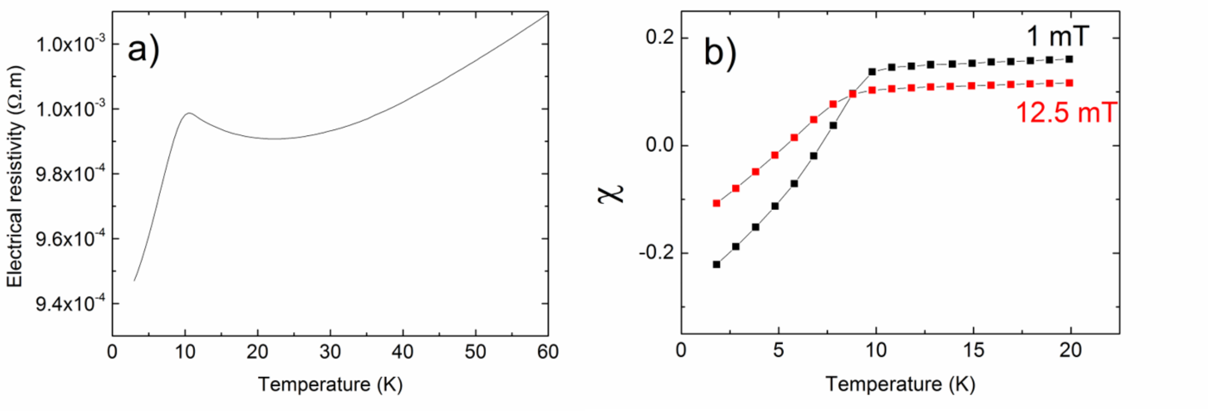

Supplementary Figure 16. a) Electrical resistivity of the compacted $LaFeSiF_{0.18}$ powder (a=4.03 Å and c=8.09 Å), presented in the synthesis part of the article. b) Magnetic susceptibility of the same powder sample measured in ZFC conditions at 1 and 12.5 mT. At 1mT, the superconducting volume of the sample amounts to 40%. A less sharp superconducting transition at 10K is observed for the resistivity but fails to drop to zero due to the poor compaction of the powder. Magnetic susceptibility displays a transition at the same temperature with a superconducting volume reaching ≈ 40%.

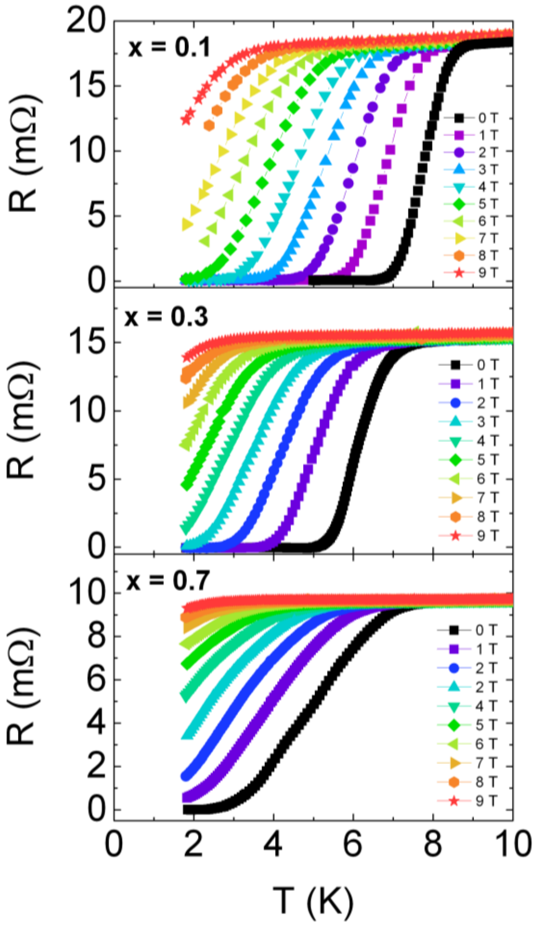

Supplementary Figure 17. Temperature dependence of the electrical resistance of the $LaFeSiF_{0.10}$ (upper panel), the $LaFeSiF_{0.30}$ (middle panel) and the $LaFeSiF_{0.70}$ (bottom panel) crystals at magnetic fields ranging from 0 to 9T.

Supplementary Notes 4

Hall resistivity measurements: In order to get more insight on the nature of the charge transport and to correlate the fluorine doping level to effective hole or electron doping, we have performed Hall coefficient measurements. The Hall effect of the sample with x = 0.7 could not be determined reliably due to its small size and shape. For the fluorinated samples with x=0.1 and x=0.3 ρ_{xy} is seen to be linear in field, as expected for a one band metal. At 10K, just above the superconducting transition, R_H is found to be positive which implies that hole-like carriers dominate the charge transport at low temperature. The magnitude of R_H is of the order of 10^{-10} m^3/C, which is one order of magnitude smaller than most of the iron-based superconductors (IBSC). Assuming a one band model, the carrier density n_H, given by $n_H = 1/eR_H$ would amount to an unreasonably large value: between 2 and 1.7 holes per Fe atom for x = 0.1 and x = 0.3 respectively. We are therefore led to conclude that a one band model is not adequate to describe the system and a multi-band model has rather to be used, in good agreement with our band structure calculation.

This represents the first result of the Hall effect measurement: the magnitude of R_H is very small, one order smaller than most of IBSC in the paramagnetic state. The second result concerns the strong temperature dependence of the Hall effect: it is seen to be highly non-trivial, with a sign change occurring at 35 K (20 K) for x = 0.1 (x = 0.3), as can be seen in Supplementary Figure 18. Interestingly, although R_H of LaFeSi displays a low temperature Hall coefficient with the same order of magnitude as that of the fluorinated samples, its temperature dependence is strikingly different: it is almost constant between 300 K and 10 K. Our band structure calculations are able to predict the correct sign and order of magnitude for the low temperature limit of R_H of LaFeSi and the fluorinated compounds: $R_H^{th} \sim 10^{-10}$ m^3/C.

It is instructive to note that the non-trivial temperature dependence and the low magnitude of R_H in the superconducting, fluorine doped sample are shared by another IBSC: the chalcogenide FeSe in its high temperature phase (above the nematic transition). FeSe is a system that has been thoroughly investigated using different experimental probe such as ARPES, quantum oscillation and magnetotransport. ARPES and quantum oscillations have been used to constrain the interpretation of the small value and the non-trivial temperature dependence of the Hall coefficient as being due to the compensated nature of the carriers,with equal hole (n_h) and electron (n_e) concentration and mobilities varying with temperature[5].

Given these similitudes, we are led to conclude that the fluorinated samples are best described as compensated metals, which precludes the determination of the evolution of carrier concentration with fluorine doping.

Finally, we note that the Hall coefficient of FeSe, which absolute value and temperature dependence are similar to that of $LaFeSiF_x$ can be interpreted as being due to orbital differentiation in Hund metals as originally proposed for the multi band superconductor Sr_2RuO_4[6].

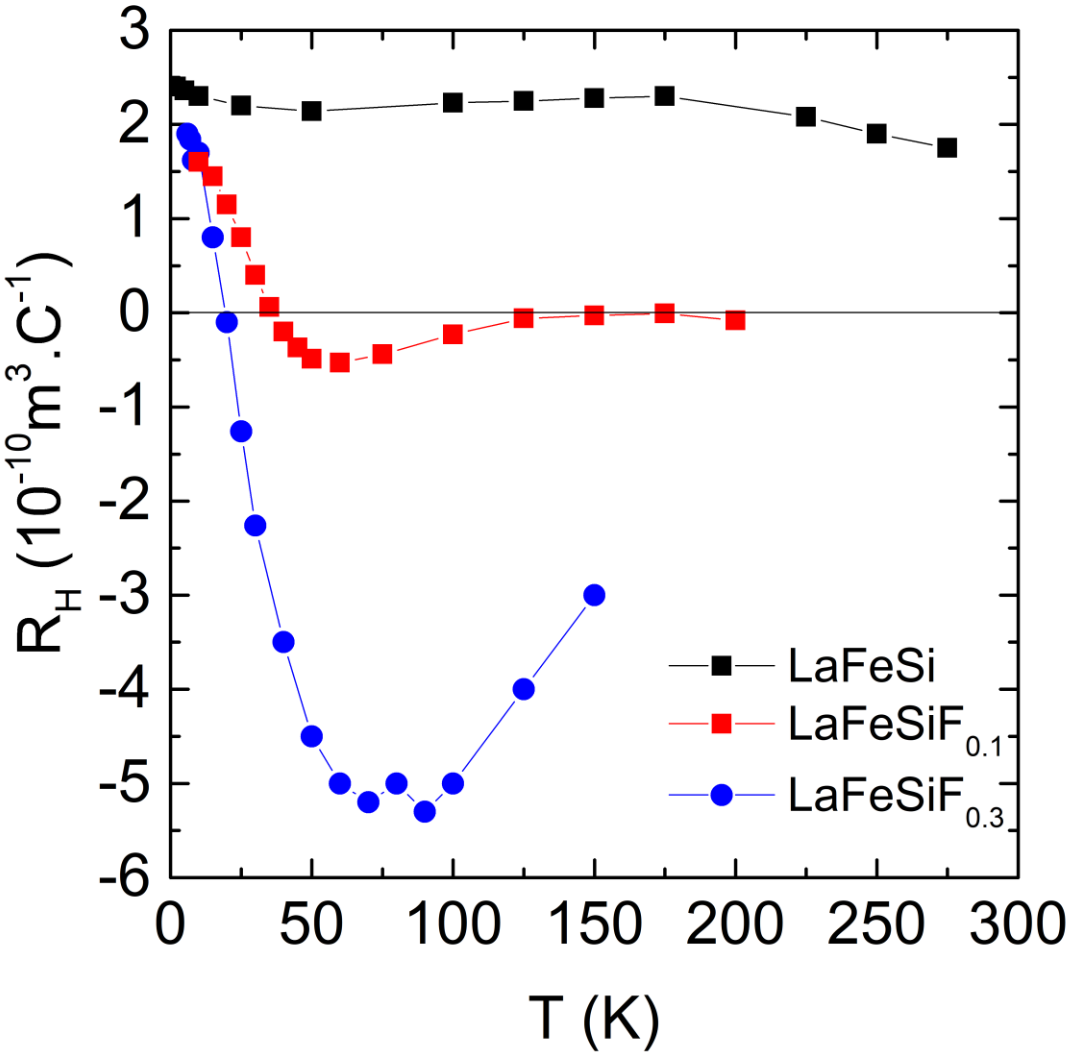

Supplementary Figure 18. Temperature dependence of the Hall resistivity of LaFeSi, $LaFeSiF_{0.1}$ and $LaFeSiF_{0.3}$ single crystals measured at 9T.

Supplementary Notes 5

Heat capacity measurements: In order to get more information on the superconducting properties as well as on the density of state at E_F, we have performed additional heat capacity measurements. The total heat capacity of the sample can be written as: $C = \gamma T + \beta T^3$ where γ corresponds to the electronic contribution to the specific heat (proportional to the DOS at E_F) and β corresponds to the phononic part. The Sommerfeld Coefficient γ can be extracted by plotting $\frac{C}{T}\ vs\ T^2$ and fitting the linear in T^2 contribution from the phonons, as presented in Supplementary Figure 19. For the x = 0.3 powder sample, this procedure yields γ = 12.5 mJ.mol^{-1}K^{-2}. This experimentally determined value can be compared to the theoretical value $\gamma^{th.}$ = 6.5 mJ.mol^{-1}K^{-2} estimated using the density of states calculated for LaFeSiF (3 states/eV/f.u., Supplementary Figure 23). Although the experimental and the theoretical value of γ are of the same order of magnitude, the experimental one is found to be significantly higher. This higher experimental value may be explained by the presence of electronic correlations that are not taken into account in the band structure calculation.

Below ~7 K (50 K^2), a slight upward deviation from the T^2 fit can be resolved. This upward deviation reaches a maximum at 4 K and then decreases with temperature, eventually crossing the T^2 fit at 2.8 K. As the temperature is further lowered, C_p/T continues to drop. It is tempting to ascribe this anomaly to a phase transition associated to superconductivity. In order to remove the phonon specific heat, we have subtracted the T^2 fit to the raw data of C_p/T, the result being displayed in Supplementary Figure 20. Clearly, this anomaly, which onsets below 7 K does not follow the sharp increase at T_c expected for a conventional BCS superconductor but rather a broad hump. Indeed, the height of the hump divided by gamma only amounts to 17%, which is low for a conventional superconductor but a value of 20% is commonly reported in 1111 IBSC. The presence of a R = 0 state, the observation of a large diamagnetic signal (85% shielding fraction for the x = 0.3 sample) and an anomaly in specific heat whose magnitude is compatible with what is observed in 1111 IBSC leads us to conclude that the sample exhibits bulk superconductivity. The broadness of the hump may be related to sample inhomogeneity and also to the fact that we are measuring a powder sample.

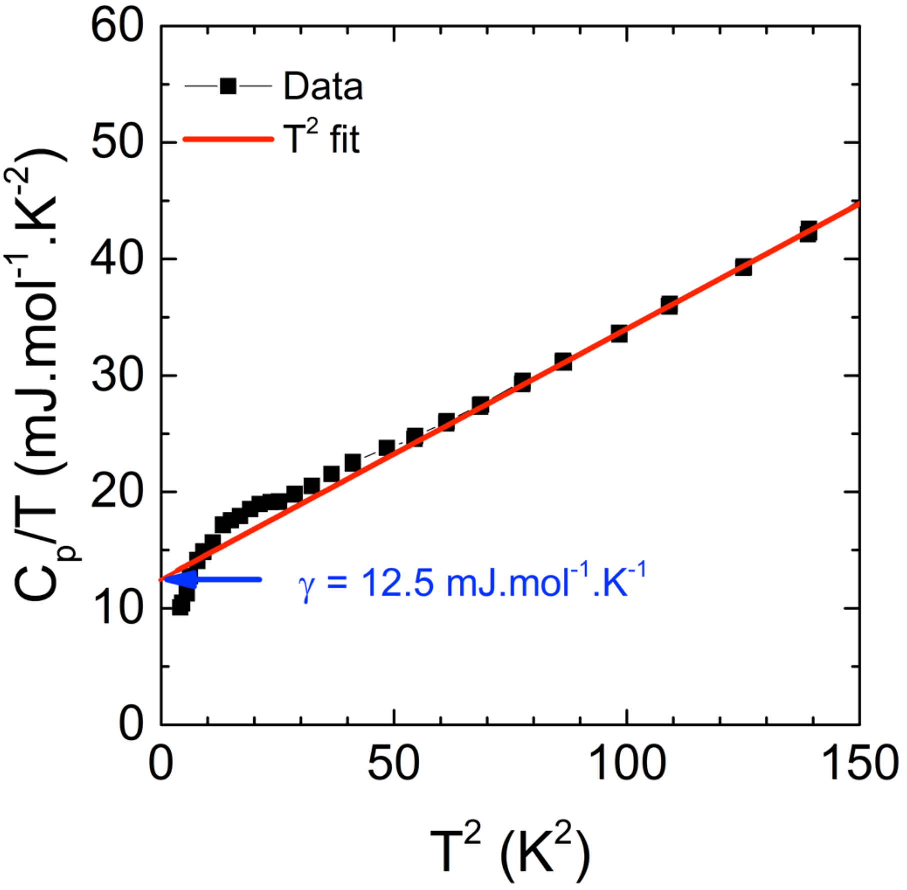

Supplementary Figure 19. Heat capacity divided by the temperature plotted against the temperature squared. The red line represents the T^2 fit of the data above T = 7 K.

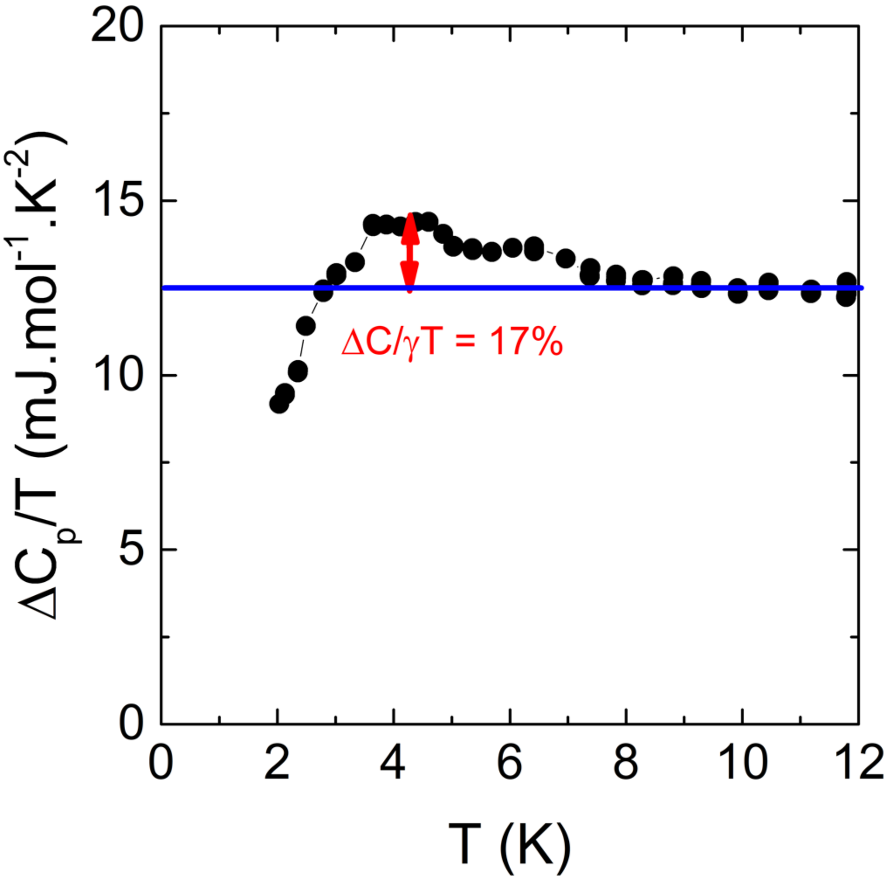

Supplementary Figure 20. Variation of the electronic heat capacity ΔCp/T as a function of temperature. The blue line represents the constant electronic contribution above T_c.

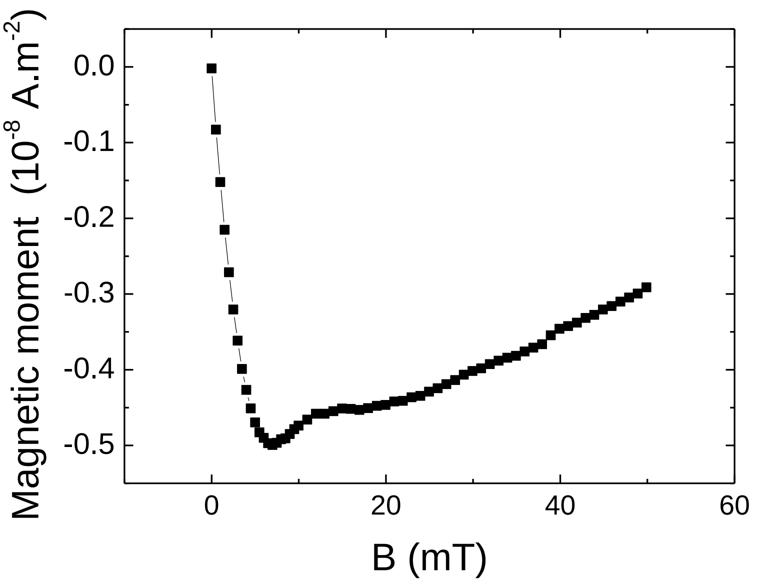

Supplementary Figure 21. Magnetic field dependence of the magnetic moment of the $LaFeSiF_{0.1}$ crystal measured up to 50mT, with field applied along the c-axis of the crystal.

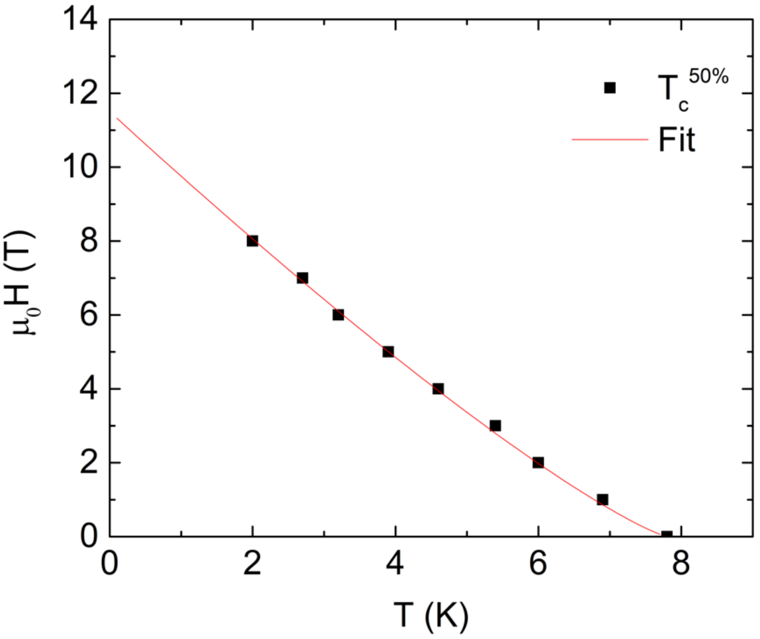

Supplementary Figure 22. Magnetic field – temperature phase diagram of $LaFeSiF_{0.1}$ enabling the determination of H_{c2} ($T \rightarrow 0$).The values of the superconducting transition temperatures measured at different magnetic field represent $T_c^{50\%}$, as defined as the temperature at which the resistance equals 50% of its value at 10K (black symbols). In order to extract the upper critical field H_{c2} the data have been fitted to a Ginzburg Landau two-band model (red curve) in order to capture the positive curvature yielding a value of H_{c2} ($T \rightarrow 0$) = 11.5T. This method has been successfully used for the determination of H_{c2} in the case of non-magnetic borocarbides[7].

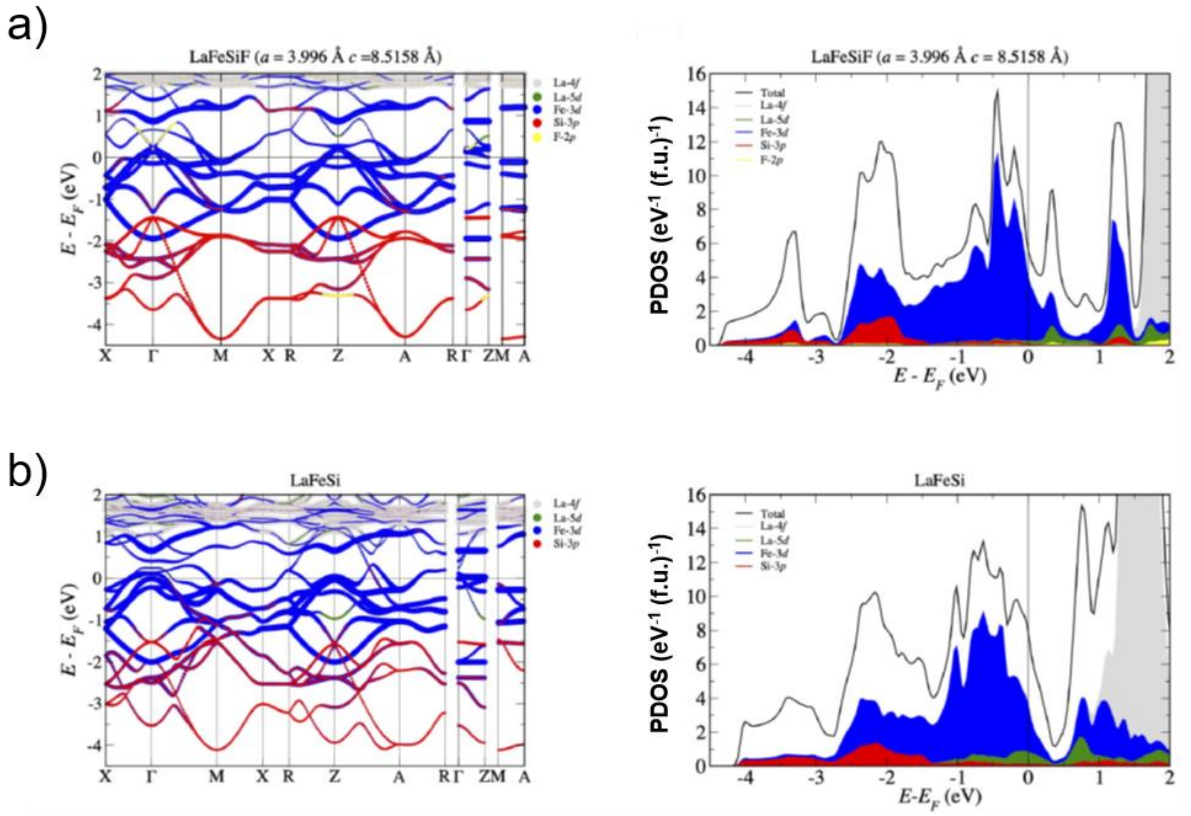

Supplementary Figure 23. Electronic band structure (left) and PDOS (right) computed for LaFeSiF (a) and LaFeSi (b) with the structural parameters determined experimentally for the x = 0.85 fluorinated silicide and the x = 0 precursor respectively.

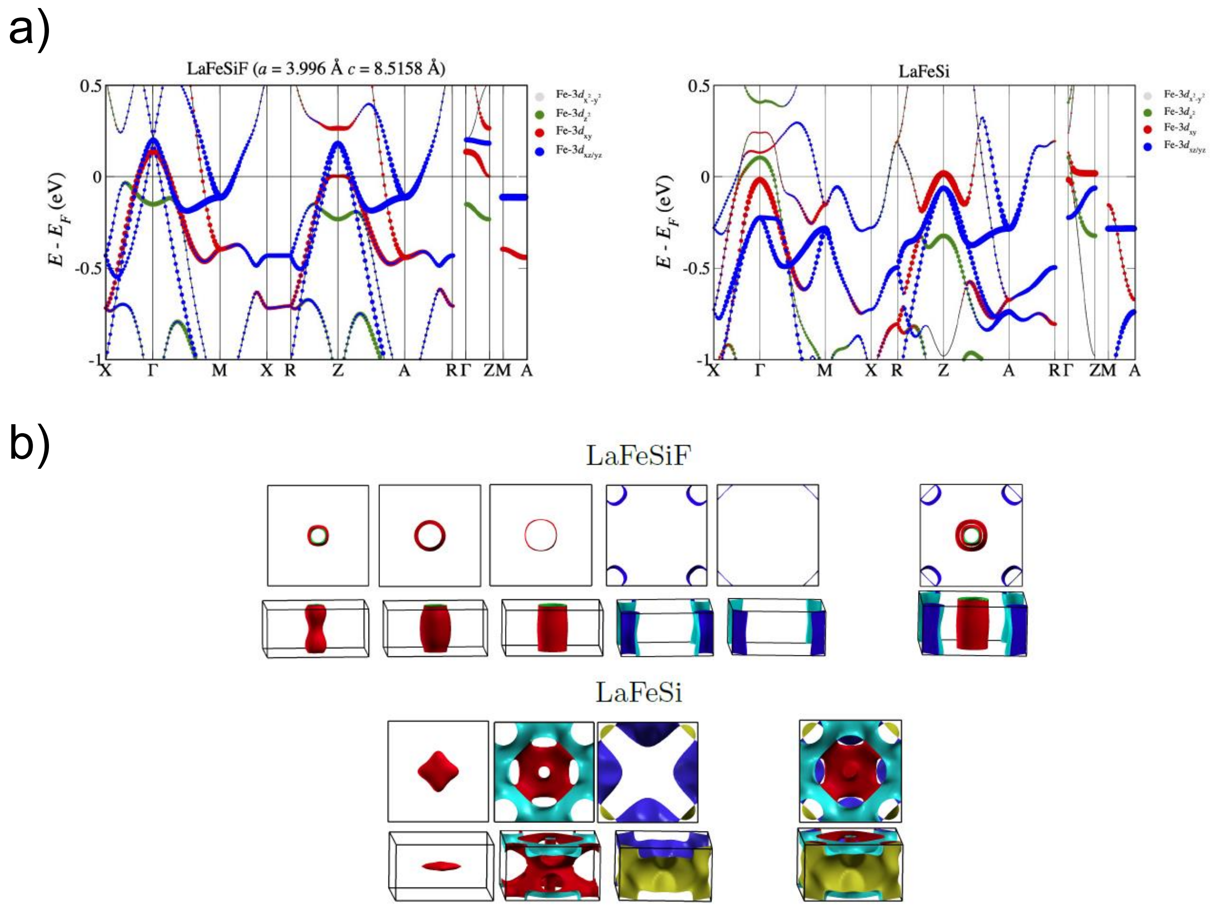

Supplementary Figure 24. (a) Electronic band structure computed for LaFeSiF (left) and LaFeSi (right) with the structural parameters determined experimentally for the x = 0.85 fluorinated silicide and the x = 0 precursor respectively. (b) Top and perspective views of the different sheets (left) and overall Fermi surface (right) computed for LaFeSiF and LaFeSi with the above parameters. The comparison with the supercell calculation shown in the main text reveals that the outer electron FS of LaFeSiF$_{7/8}$ is strongly convoluted with non-stoichiometry effects.

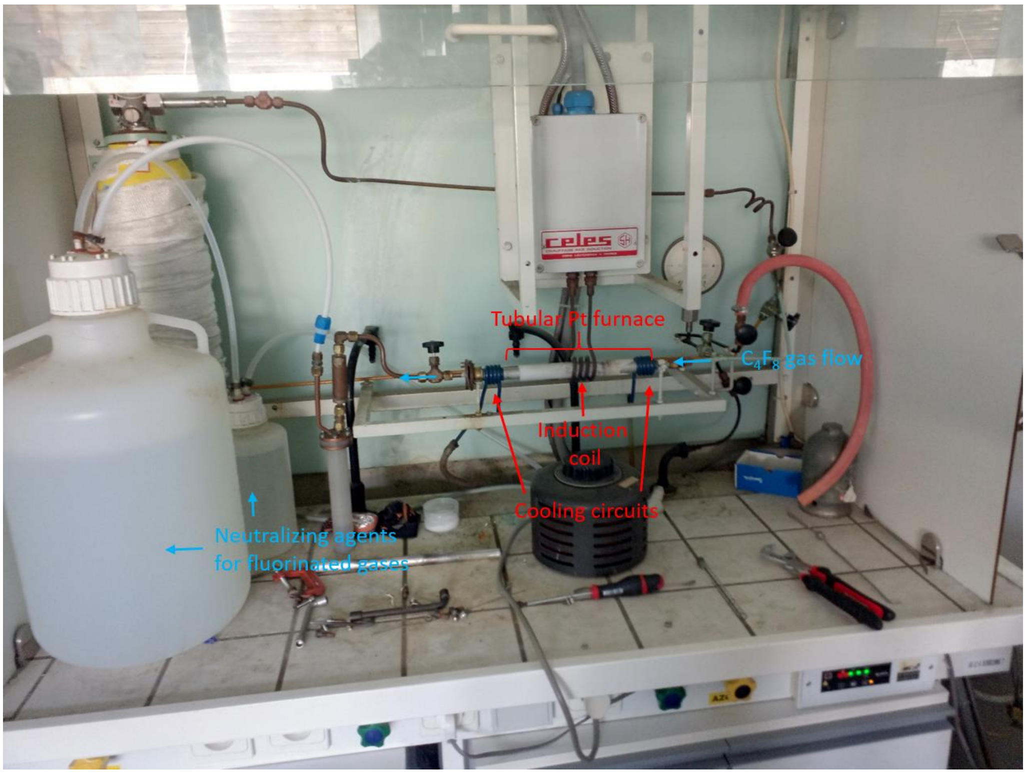

Supplementary Figure 25. Set-up used for fluorination using C_4F_8.The furnace itself is made of Pt and heated inductively at the center by an induction coil. The temperature is controlled manually by adjusting the power source, and was calibrated prior to the experiments. The furnace is cooled at both ends to condensate volatile species and the gas outlet pipe are plunged into tank of harmful fluorinated gas neutralizing agents (liquid potash or soda). The whole set-up needs to be kept inside a hood.

Supplementary References

1. De Marco, R. *et al.* XPS studies of the fluoride ion-selective electrode membrane LaF3: Evidence for a gel layer on the surface. *Surf. Interface Anal.* **14**, 463–468 (1989).
2. Xie, Q. *et al.* A novel photocatalyst LaOF: Facile fabrication and photocatalytic hydrogen production. *Catal. Commun.* **27**, 21–25 (2012).
3. Schmidt, S. *et al.* Reactive high power impulse magnetron sputtering of CFx thin films in mixed Ar/CF4 and Ar/C4F8 discharges. *Thin Solid Films* **542**, 21–30 (2013).
4. Beamson, G. & Briggs, D. *High Resolution XPS of Organic Polymers: The Scienta ESCA300 Database*. (Wiley, 1992).
5. Watson, M. D. *et al.* Dichotomy between the Hole and Electron Behavior in Multiband Superconductor FeSe Probed by Ultrahigh Magnetic Fields. *Phys. Rev. Lett.* **115**, 027006 (2015).
6. Zingl, M., Mravlje, J., Aichhorn, M., Parcollet, O. & Georges, A. Hall coefficient signals orbital differentiation in the Hund’s metal Sr2RuO4. *npj Quantum Mater. 2019 41* **4**, 1–6 (2019).
7. Drechsler, S.-L., Rosner, H., Shulga, S. V & Eschrig, H. Superconducting Transition Metal Borocarbides. in *High-Tc Superconductors and Related Materials: Material Science, Fundamental Properties, and Some Future Electronic Applications* (eds. Drechsler, S.-L. & Mishonov, T.) 167–184 (Springer Netherlands, 2001). doi:10.1007/978-94-010-0758-0_7
